# A new Fermatean fuzzy Spearman-like correlation coefficient and its application in evaluating insecurity problem via multi-criteria decision-making approach

**DOI:** 10.1016/j.heliyon.2024.e40403

**Published:** 2024-11-15

**Authors:** Paul Augustine Ejegwa, Nasreen Kausar, Nezir Aydin, Yuming Feng, Oludolapo Akanni Olanrewaju

**Affiliations:** a Department of Mathematics, Joseph Sarwuan Tarka University, Makurdi, Nigeria; b Department of Mathematics, Faculty of Arts and Science, Yildiz Technical University, Esenler 34220, Istanbul, Turkiye; c College of Science and Engineering, Hamad Bin Khalifa University, Doha, Qatar; d Department of Industrial Engineering, Yildiz Technical University, Besiktas, 34349 Istanbul, Turkiye; e Key Laboratory of Intelligent Information Processing and Control, Chongqing Three Gorges University, Chongqing 404100, China; f Department of Industrial Engineering, Durban University of Technology, Durban 4001, South Africa

**Keywords:** Insecurity assessment, Security crises, Fermatean fuzzy sets, Correlation coefficient, Multi-criteria decision-making, North central region of Nigeria

## Abstract

The problem of insecurity is a global crisis with adverse effects on lives and properties. Fermatean fuzzy correlation coefficient is a dependable method for handling imprecision, which is the main bottleneck of insecurity assessment. A number of Fermatean fuzzy correlation coefficient methods have been developed. Based on Spearman's correlation coefficient, an innovative Fermatean fuzzy correlation coefficient method is built to enhance trustworthy insecurity assessment. The existing Fermatean fuzzy correlation coefficient methods are evaluated, and their shortcomings are identified in order to validate the construction of a new Fermatean fuzzy correlation coefficient method. The drawbacks of the existing methods lead us into building a new Fermatean fuzzy correlation coefficient method by using the Spearman's correlation coefficient approach, which has the potential of overcoming the drawbacks of the existing Fermatean fuzzy correlation coefficient methods. In addition, some theoretical findings are provided to support the strength of the novel Fermatean fuzzy correlation coefficient method and it is shown that the new method satisfies the Fermatean fuzzy correlation coefficient requirements. Furthermore, the novel Fermatean fuzzy correlation coefficient method is applied to assess the insecurity situation in the North-Central Region of Nigeria to furnish intended tourists with relevant travel advice. To demonstrate the inherent significance of the novel Fermatean fuzzy correlation coefficient method, we compare its effectiveness to that of the extant Fermatean fuzzy correlation coefficient methods. The results of the comparison show the superiority of the novel Fermatean fuzzy correlation coefficient method over the existing ones in terms of reliability, consistency, precision and compliance with the Fermatean fuzzy correlation coefficient axioms. Ultimately, it is discovered that the new method can effectively address hesitations related to insecurity assessment.

**Table:** 

Abbreviations and their meanings.	
Abbreviations	Meanings

NCRN	North Central Region of Nigeria
FFSs	Fermatean Fuzzy Sets
IFSs	Intuitionistic Fuzzy Sets
PFSs	Pythagorean Fuzzy Sets
FFCC	Fermatean Fuzzy Correlation Coefficient
FFCCMs	Fermatean Fuzzy Correlation Coefficient Methods
MCDM	Multi-Criteria Decision-Making
TOPSIS	Technique for Order of Preference by Similarity to Ideal Solution
IFHM	Intuitionistic Fuzzy Hesitation Margin
PFHM	Pythagorean fuzzy hesitation margin
FFHM	Fermatean fuzzy hesitation margin
MDs	Membership Degrees
NMDs	Non-Membership Degrees
FCT	Federal Capital Territory
FFDM	Fermatean Fuzzy Decision Matrix
NFFDM	Normalized Fermatean Fuzzy Decision Matrix
WNFFDM	Weighted Normalized Fermatean Fuzzy Decision Matrix
CC	Cost Criterion
BC	Benefit Criteria
FFNs	Fermatean Fuzzy Numbers
PIS	Positive Ideal Solution
NIS	Negative Ideal Solution
AI	Artificial Intelligence
KBS	knowledge-Based System
LVs	Linguistic Variables
EH	Extremely High
VVH	Very Very High
VH	Very High
H	High
MH	Medium High
M	Medium
ML	Medium Low
L	Low
VL	Very Low
VVL	Very Very Low
EL	Extremely Low

## 1 Introduction

Security and safety are main subjects of global concern. The term security problems happen due to the breakdown of law and order causing loss of properties and lives. It happens in diverse ways like armed robbery, terrorisms, banditry, kidnapping for ransom, communal clashes, and militancy, among others. Everyone endeavors to live a secure life, and as such, extensive efforts have being made to guarantee individual safety [1]. Nigeria has been plagued with security crises. Northern Nigeria consists of three regions that have faced insecurity for over a decade [2], [3]. One of the regions in the Northern Nigeria that is engulfed in insecurity is the NCRN. Some security experts alleged that the case of insecurities in the north is due to its geographical terrain and the ungoverned spaces [4], [5]. Numerous minority ethnic groups reside in the NCRN, which is made up of six states in addition to Abuja, the nation's capital. There are more than 200 different languages spoken in the NCRN. The NCRN is experiencing a multifaceted crisis stemming from long-standing conflicts between ethnic and religious communities, as well as assaults by armed bandits and criminal organizations, including highway robberies and kidnappings [6]. The increase in armed bandits in the NCRN has resulted in a security crisis that is epitomized by maimings, kidnappings, killings, cattle rustling, population displacement, and disruption of socio-economic activities [7]. Both the government and the populace are beginning to express concern about the uncertain atmosphere these events have brought to the NCRN.

Given the abundance of intricately linked causative factors and dimensions inherent in the threats posed by this development, it is clear that armed banditry in the NCRN is more than just a pastoralist insurgency [6], [8]. Experts have itemized the following as some causes of insecurity in the NCRN, viz. ethno-religious conflicts/terrorism, farmer-herder clashes due to open grazing practice (i.e., clashes owing to the use of land, water, and grazing routes), banditry and kidnapping due to poverty arisen from pervasive material inequalities and corruption, struggles/mayhems due to mineral resources, weak/complicit security architecture, porous borders, and political agitations [4], [6], [7]. Zubairu [4] suggested that provision of affordable education, job creation and youth empowerment, effective control of light or small arms and weapons, improve border security and policing, and strengthening of the criminal justice system could help in abating insecurities in the NCRN enclaves. Many studies have been carried out to identify the most insecure state in the NCRN to no avail due to the uncertainty, unpredictability, and fuzziness of the insecurity situations in the region. Thus, a reliable evaluation/assessment of security crises can be achieved by the deployment of advanced soft computing tools like the FFS [9], which is proficient in handling fuzziness and imprecision.

### 1.1 Review of literature

The theory of FFSs generalizes the fuzzy sets, IFSs, and PFSs for an effective result. The fuzzy set [10] theory extended set theory by the inclusion of membership grade, which is defined as a function from the classical set to the unit interval. Nonetheless, fuzzy set can only handle uncertainty with an inability to tackle imprecision because of the exclusion of the hesitation margin. The term hesitation margin is the grade that describes the delay or uncertainty involves in making a decision. By incorporating the hesitation margin with the membership grade alongside the non-membership grade, Atanassov [11] introduced the IFS theory where the non-membership grade is not necessary 1 minus the membership grade and the hesitation margin is defined as 1 minus the aggregate of the membership and non-membership grades. In addition, the addition of both membership and non-membership grades must not exceed 1. By using the IFSs' design, several authors have discussed many practical problems. Zhou et al. [12], Zeng et al. [13] and Ejegwa et al. [14] discussed the applications of IFSs in the problems of pattern recognition using different approaches, and the use of IFSs in medical science was discussed in [15], [16]. IFSs were used to discuss decision-making via intuitionistic fuzzy (IF) aggregation operators [17], [18], Ejegwa et al. [19] discussed decision-making with IF distance functions, Zhang et al. [20] used ranking method of IFSs to discuss threat assessment, and the utilization of IFSs in students' admission and sundry practical problems were discussed in [21], [22].

However, in case the aggregate of the membership and non-membership grades exceeds 1, Yager [23] introduced a set called the PFSs which is the expansion of IFSs. Because of the wider scope of PFSs, the idea has been extensively used to discuss many practical problems like decision-making [24], [25], football analysis [26], and pattern recognition [27]. Wu et al. [28] and Zhang [29] showed the practical application of PFSs in decision-making based on similarity and distance metrics, Zhang and Xu [30] discussed TOPSIS method in PFS setting, and some decision-making problems were presented based on correlation between PFSs [31], [32]. More so, the applications of other higher order fuzzy sets have been discussed in the evaluation of financial logistics enterprises and public health emergencies [33], [34]. But, the conspicuous setback of the system of PFSs is that, the squares of the sum of the membership and non-membership grades must not be more than 1. In the event it exceeds 1, to cope with such scenarios, the concept of FFSs was introduced by Senapati and Yager [9] to model situations where the sum of the square of both non-membership and membership grades exceeds 1. This shows that the scope of applications of FFSs is wider than that of both IFSs and PFSs, respectively. Consequently, the concept of FFSs has been widely applied in decision-making problems. In [35], [36], MCDM method was discussed using Fermatean fuzzy geometric operators, Aydin [37] applied theories of FFSs to MCDM, Akram et al. [38] discussed a MCDM method via complex FFSs, and a process of students' ranking via Fermatean fuzzy information was discussed in [39]. In addition, diagnosis of disease [40], [41], pattern recognition [42], [43], and selection process and clustering [44] have been discussed using various theories of FFSs. Furthermore, FFSs have been applied to discuss renewable energy sources [45], [46], waste management [47], sustainable urban transport [48], and decision-making [49]. In addition, similarity and distance metrics of FFSs have been used to discuss some decision-making methods [50], [51], [52], admission process [53], and clusterings [54].

In a way to show robust applications of uncertain systems, the idea of correlation coefficient has been stretched to uncertain environments. To start with, correlation coefficient is a tool used to estimate the relationship between two classical or fuzzy data. The applications of the concept have been explored in engineering, medical science, machine learning, image segmentations, etc. Numerous techniques of fuzzy correlation coefficient [55], intuitionistic fuzzy correlation coefficient [56], [57], [58], [59], and Pythagorean fuzzy correlation coefficient [60], [61], [62], [63] have been researched and applied to various areas of application cutting across medical sciences, decision-making, etc. Because of the limitations of fuzzy sets, IFSs, and PFSs as established, the idea of correlation coefficient analysis has been stretched to Fermatean fuzzy domain. First and foremost, Kirisci [64] introduced the idea of FFCC by developing two FFCCMs based on Pearson's correlation approach, and discussed their application in disease diagnosis. The FFCCMs are defective because they cannot estimate the FFCC with interchanging zero membership and non-membership grades. Bhatia et al. [65] developed a system of FFCC from a statistical viewpoint defined within [−1,1], and used it to discuss pattern analysis and supplier selection. This method is not dependable whenever the set is singleton or if each element of the set in the FFSs has the same membership and non-membership grades. Furthermore, two FFCCMs were presented in [66] and used to discuss the principle of disease diagnosis. One of the FFCCMs shared the same setback with the approaches in [64], while the other yields less precise result as the cardinality of the set becomes larger. Gouli et al. [67] presented a Pearson-like FFCCM with application in the selection of electric car based on MCDM. The method is not dependable whenever each element of the FFSs has the same membership and non-membership grades just like the approach in [65]. In addition, Amman et al. [68] presented a FFCCM by using Spearman rank correlation coefficient with application to TOPSIS. The approach is a direct extension of the correlation coefficient under IFSs [59] and PFSs [63], and it yields unrealistic result whenever the underlying set for the FFSs is singleton. Again, the method cannot properly curb uncertainty because the ranks of the data are presented as discrete values.

### 1.2 Motivation and contributions

Several decision-making problems have been investigated based on FFCCMs [64], [65], [66], [67], [68]. The choice of FFSs over either IFSs or PFSs in evaluating security crises is due to the capacity of FFSs to handle complex cases of imprecision in evaluation and assessment. From the reviewed works on FFCCMs [64], [65], [66], [67], [68], the motivation for the construction of a new FFCCM is justifiable because of the stated weaknesses of the existing FFCCMs. Although some security experts have evaluated the security crises in the NCRN [69], [70], [71], [72], they do not evaluate and assess the crises using soft computing approaches like FFCCMs. Hence, the outcomes of their investigations cannot be reliable because the fuzziness of security crises is not captured. Motivated by the research gap in security evaluation/assessment and the weaknesses of the existing FFCCMs, this work presents a new FFCCM using the Spearman's correlation model and incorporating the complete parameters of FFSs to evaluate the security crises within the NCRN. Succinctly speaking, this study is aimed at presenting a new FFCCM and its application in the assessment of the level of insecurity in the NCRN based on TOPSIS method. The new FFCCM is constructed by taking into account the design of the Spearman's correlation coefficient, the three defining attributes of FFSs, and the influence of the weights of the elements of the FFSs. All these are considered to enhance consistency, precision, and reliability. The contributions of the works are delineated as follows:

• analyzing the existing FFCCMs [64], [65], [66], [67], [68] to showcase their weakness and level of agreement with the FFCC conditions.
• constructing a novel FFCCM which can fix all the drawbacks of the existing FFCCMs.
• presenting the theoretical properties of the novel FFCCM to validate its construction.
• discussing the practicability of the novel FFCCM in the assessment of insecurity in the NCRN based on TOPSIS method.
• showcasing the strength of the novel FFCCM in comparison to the existing FFCCMs [64], [65], [66], [67], [68] in literature.

The remaining part of the paper is made up of the following sections: Section 2 covers the basics of FFSs and the existing FFCCMs [64], [65], [66], [67], [68], Section 3 presents the new FFCCM and its theoretical properties with numerical verifications, Section 4 discusses the practical usefulness of the novel FFCCM in the assessment of insecurity in the NCRN using TOPSIS method together with comparative analyses, and Section 5 presents the conclusion and a number of recommendations.

## 2 Preliminaries

This section covers the basics of FFSs and the definitions FFCC as a precursor to the introduction of the new FFCCM. In this paper, we refer to $Z=\{z_{1},z_{2},\cdots ,z_{N}\}$, $N\in Z^{+}$ as the underlying set.

Definition 1 **[9], [11], [23]**

Consider a set-structure $ℑ=\{(z_{j},{ℑ}_{m}(z_{j}),{ℑ}_{n}(z_{j}))|z_{j}\in Z\}$, where ${ℑ}_{m},{ℑ}_{n}:Z\to [0,1]$ are functions which represent both the membership and non-membership grades, respectively. Then, ℑ is called an IFS if (1) holds:

(1) $${ℑ}_{m}(z_{j})+{ℑ}_{n}(z_{j})\leq 1,$$

and ${ℑ}_{h}(z_{j})=1-{ℑ}_{m}(z_{j})-{ℑ}_{n}(z_{j})$ is the IFHM of ℑ in *Z* such that ${ℑ}_{m}(z_{j})+{ℑ}_{n}(z_{j})+{ℑ}_{h}(z_{j})=1$.

In addition, ℑ becomes a PFS if (2) holds:

(2) $${ℑ}_{m}^{2}(z_{j})+{ℑ}_{n}^{2}(z_{j})\leq 1,$$

and ${ℑ}_{h}(z_{j})=\sqrt{1-{ℑ}_{m}^{2}(z_{j})-{ℑ}_{n}^{2}(z_{j})}$ is the PFHM of ℑ in *Z* such that ${ℑ}_{m}^{2}(z_{j})+{ℑ}_{n}^{2}(z_{j})+{ℑ}_{h}^{2}(z_{j})=1$.

Finally, ℑ is called a FFS if (3) holds:

(3) $${ℑ}_{m}^{3}(z_{j})+{ℑ}_{n}^{3}(z_{j})\leq 1,$$

and ${ℑ}_{h}(z_{j})=\sqrt[{3}]{1-{ℑ}_{m}^{3}(z_{j})-{ℑ}_{n}^{3}(z_{j})}$ is the FFHM of ℑ in *Z* such that ${ℑ}_{m}^{3}(z_{j})+{ℑ}_{n}^{3}(z_{j})+{ℑ}_{h}^{3}(z_{j})=1$.

Certainly, the scope of a FFS is wider than the scopes of an IFS and a PFS, respectively. If the family of the IFSs is represented by IFS(Z), the family of the PFSs is represented by PFS(Z), and the family of the FFSs is represented by FFS(Z), then we have the inclusion IFS(Z)⊂PFS(Z)⊂FFS(Z).

Now, the basic properties of the FFSs are delineated in Definition 2.

Definition 2 **[9]**

Assume that ℑ and ℜ are FFSs in *Z*, then we define the following:

(i) ${ℑ}^{c}=\{(z_{j},{ℑ}_{n}(z_{j}),{ℑ}_{m}(z_{j}))|z_{j}\in Z\}$, ${ℜ}^{c}=\{(z_{j},{ℜ}_{n}(z_{j}),{ℜ}_{m}(z_{j}))|z_{j}\in Z\}$.
(ii) $ℑ\cup ℜ=\{(z_{j},\max\{{ℑ}_{m}(z_{j}),{ℜ}_{m}(z_{j})\},\min\{{ℑ}_{n}(z_{j}),{ℜ}_{n}(z_{j})\})|z_{j}\in Z\}$.
(iii) $ℑ\cap ℜ=\{(z_{j},\min\{{ℑ}_{m}(z_{j}),{ℜ}_{m}(z_{j})\},\max\{{ℑ}_{n}(z_{j}),{ℜ}_{n}(z_{j})\})|z_{j}\in Z\}$.
(iv) ℑ=ℜ iff ${ℑ}_{m}(z_{j})={ℜ}_{m}(z_{j})$ and ${ℑ}_{n}(z_{j})={ℜ}_{n}(z_{j})$ ∀ $z_{j}\in Z$.
(v) ℑ⊆ℜ iff ${ℑ}_{m}(z_{j})\leq {ℜ}_{m}(z_{j})$ and ${ℑ}_{n}(z_{j})\geq {ℜ}_{n}(z_{j})$ ∀ $z_{j}\in Z$.

The concept of FFCC has been defined from two perspectives presented as follows:

Definition 3 **[66]**

The coefficient of correlation between two FFSs ℑ and ℜ in the set $Z=\{z_{1},z_{2},\cdots ,z_{N}\}$, $N\in Z^{+}$ is a function

ϒ:FFS(Z)×FFS(Z)→[−1,1]or[0,1]

represented by ϒ(ℑ,ℜ), which satisfies:

C1 ϒ(ℑ,ℜ)∈[−1,1] or [0,1],
C2 ϒ(ℑ,ℜ)=1 iff ℑ=ℜ,
C3 ϒ(ℑ,ℜ)=ϒ(ℜ,ℑ).

From the FFCC conditions, a strong correlation between ℑ and ℜ is indicated by ϒ(ℑ,ℜ) approaching 1, and ϒ(ℑ,ℜ) approaching −1 or 0 indicates a weak correlation between the FFSs. In addition, ϒ(ℑ,ℜ)=−1 or 0 denotes negative perfect correlation or zero correlation and ϒ(ℑ,ℜ)=1 expresses a perfect positive correlation between the FFSs.

### 2.1 Existing FFCCMs

Assume ℑ and ℜ are FFSs defined in $Z=\{z_{1},z_{2},\cdots ,z_{N}\}$, N<∞. Then, Kirisci [64] constructed the following FFCCMs in (4) and (5):

(4) $${ϒ}_{1}(ℑ,ℜ)=\frac{\rho (ℑ,ℜ)}{\sqrt{\rho (ℑ,ℑ)\rho (ℜ,ℜ)}},$$

(5) $${ϒ}_{2}(ℑ,ℜ)=\frac{\rho (ℑ,ℜ)}{\max\{\rho (ℑ,ℑ),\rho (ℜ,ℜ)\}},$$

where the methods' parameters are defined in (6):

(6) $$\begin{array}{c} \rho (ℑ,ℜ)=\sum_{j=1}^{N}({ℑ}_{m}^{3}(z_{j}){ℜ}_{m}^{3}(z_{j})+{ℑ}_{n}^{3}(z_{j}){ℜ}_{n}^{3}(z_{j})+{ℑ}_{h}^{3}(z_{j}){ℜ}_{h}^{3}(z_{j}))\\ \rho (ℑ,ℑ)=\sum_{j=1}^{N}({ℑ}_{m}^{6}(z_{j})+{ℑ}_{n}^{6}(z_{j})+{ℑ}_{h}^{6}(z_{j}))\\ \rho (ℜ,ℜ)=\sum_{j=1}^{N}({ℜ}_{m}^{6}(z_{j})+{ℜ}_{n}^{6}(z_{j})+{ℜ}_{h}^{6}(z_{j})) \end{array}\}.$$

The drawback of these FFCCMs (4), (5) is that, they cannot estimate the FFCC with interchanging zero MDs and NMDs, respectively.

According to Bhatia et al. [65], a method for calculating FFCCM was introduced, which is presented in (7):

(7) $${ϒ}_{3}(ℑ,ℜ)=\frac{\rho (ℑ,ℜ)}{\sqrt{\rho (ℑ,ℑ)\rho (ℜ,ℜ)}},$$

where the method's parameters are defined in (8):

(8) $$\begin{array}{c} \rho (ℑ,ℜ)=\frac{\sum_{j=1}^{N}(d(ℑ)d(ℜ))}{N-1}\\ \rho (ℑ,ℑ)=\frac{\sum_{j=1}^{N}d^{2}(ℑ)}{N-1}\\ \rho (ℜ,ℜ)=\frac{\sum_{j=1}^{N}d^{2}(ℜ)}{N-1} \end{array}\}.$$

The deviations of the FFSs, d(ℑ) and d(ℜ) are defined in (9):

(9) $$\begin{array}{c} d(ℑ)=\sum_{j=1}^{N}(({ℑ}_{m}^{3}(z_{j})-{\bar{ℑ}}_{m}^{3})-({ℑ}_{n}^{3}(z_{j})-{\bar{ℑ}}_{n}^{3})-({ℑ}_{h}^{3}(z_{j})-{\bar{ℑ}}_{h}^{3}))\\ d(ℜ)=\sum_{j=1}^{N}(({ℜ}_{m}^{3}(z_{j})-{\bar{ℜ}}_{m}^{3})-({ℜ}_{n}^{3}(z_{j})-{\bar{ℜ}}_{n}^{3})-({ℜ}_{h}^{3}(z_{j})-{\bar{ℜ}}_{h}^{3})) \end{array}\},$$

where their means, $\bar{ℑ}$ and $\bar{ℜ}$ are in (10):

(10) $$\begin{array}{c} {\bar{ℑ}}_{m}=\frac{\sum_{j=1}^{N}{ℑ}_{m}(z_{j})}{N},{\bar{ℑ}}_{n}=\frac{\sum_{j=1}^{N}{ℑ}_{n}(z_{j})}{N},{\bar{ℑ}}_{h}=\frac{\sum_{j=1}^{N}{ℑ}_{h}(z_{j})}{N}\\ {\bar{ℜ}}_{m}=\frac{\sum_{j=1}^{N}{ℜ}_{m}(z_{j})}{N},{\bar{ℜ}}_{n}=\frac{\sum_{j=1}^{N}{ℜ}_{n}(z_{j})}{N}{\bar{ℜ}}_{h}=\frac{\sum_{j=1}^{N}{ℜ}_{h}(z_{j})}{N} \end{array}\}.$$

This method (7) is not dependable whenever |Z|=1 or if each element of *Z* in the FFSs has the same MD and NMD, respectively.

In addition, Ejegwa and Sarkar [66] presented two FFCCMs, the first is presented as (11):

(11) $${ϒ}_{4}(ℑ,ℜ)=\frac{\sum_{j=1}^{k}(\alpha_{j}(1-\Pi_{mj})+\beta_{j}(1-\Pi_{nj})+\gamma_{j}(1-\Pi_{hj}))}{3N},$$

where the method's parameters are defined in (12) and (13):

(12) $$\begin{array}{c} \alpha_{j}=\frac{q-\Pi_{mj}-\max\Pi_{m}}{q-\min\Pi_{m}-\max\Pi_{m}},\beta_{j}=\frac{q-\Pi_{nj}-\max\Pi_{n}}{q-\min\Pi_{n}-\max\Pi_{n}}\\ \gamma_{j}=\frac{q-\Pi_{hj}-\max\Pi_{h}}{q-\min\Pi_{h}-\max\Pi_{h}} \end{array}\},$$

for q>3, and

(13) $$\begin{array}{c} \Pi_{mj}=|{ℑ}_{m}^{3}(z_{j})-{ℜ}_{m}^{3}(z_{j})|,\Pi_{nj}=|{ℑ}_{n}^{3}(z_{j})-{ℜ}_{n}^{3}(z_{j})|\\ \Pi_{hj}=|{ℑ}_{h}^{3}(z_{j})-{ℜ}_{h}^{3}(z_{j})|\\ \min\Pi_{m}=\min_{1\leq j\leq N}\{\Pi_{mj}\},\min\Pi_{n}=\min_{1\leq j\leq N}\{\Pi_{nj}\}\\ \min\Pi_{h}=\min_{1\leq j\leq N}\{\Pi_{hj}\}\\ \max\Pi_{m}=\max_{1\leq j\leq N}\{\Pi_{mj}\},\max\Pi_{n}=\max_{1\leq j\leq N}\{\Pi_{nj}\}\\ \max\Pi_{h}=\max_{1\leq j\leq N}\{\Pi_{hj}\} \end{array}\}.$$

The FFCCM (11) is reliable in terms of efficiency and mathematical correctness but it losses accuracy as *N* increases. The second FFCCM in [66] is presented in (14):

(14) $${ϒ}_{5}(ℑ,ℜ)=\frac{\sqrt[{3}]{\rho (ℑ,ℜ)}}{\sqrt[{6}]{\rho (ℑ,ℑ)\rho (ℜ,ℜ)}},$$

where ρ(ℑ,ℜ), ρ(ℑ,ℑ), and ρ(ℜ,ℜ) are defined in (6). The FFCCM (14) is not dependable because it cannot estimate the FFCC with interchanging zero MDs and NMDs, respectively. In fact, its setback is similar to the FFCCMs in [64].

In continuation of the study of FFCCM, Gouli et al. [67] presented a Pearson-like FFCCM as seen in (15):

(15) $${ϒ}_{6}(ℑ,ℜ)=\frac{\rho_{m}(ℑ,ℜ)+\rho_{n}(ℑ,ℜ)+\rho_{h}(ℑ,ℜ)}{3},$$

where the method's parameters are defined in (16):

(16) $$\begin{array}{c} \rho_{m}(ℑ,ℜ)=\frac{\sum_{j=1}^{N}({ℑ}_{m}^{3}(z_{j})-{\bar{ℑ}}_{m}^{3})({ℜ}_{m}^{3}(z_{j})-{\bar{ℜ}}_{m}^{3})}{\sqrt{\sum_{j=1}^{N}{\left({ℑ}_{m}^{3}(z_{j})-{\bar{ℑ}}_{m}^{3}\right)}^{2}}\sqrt{\sum_{j=1}^{N}{\left({ℜ}_{m}^{3}(z_{j})-{\bar{ℜ}}_{m}^{3}\right)}^{2}}}\\ \rho_{n}(ℑ,ℜ)=\frac{\sum_{j=1}^{N}({ℑ}_{n}^{3}(z_{j})-{\bar{ℑ}}_{n}^{3})({ℜ}_{n}^{3}(z_{j})-{\bar{ℜ}}_{n}^{3})}{\sqrt{\sum_{j=1}^{N}{\left({ℑ}_{n}^{3}(z_{j})-{\bar{ℑ}}_{n}^{3}\right)}^{2}}\sqrt{\sum_{j=1}^{N}{\left({ℜ}_{n}^{3}(z_{j})-{\bar{ℜ}}_{n}^{3}\right)}^{2}}}\\ \rho_{h}(ℑ,ℜ)=\frac{\sum_{j=1}^{N}({ℑ}_{h}^{3}(z_{j})-{\bar{ℑ}}_{h}^{3})({ℜ}_{h}^{3}(z_{j})-{\bar{ℜ}}_{h}^{3})}{\sqrt{\sum_{j=1}^{N}{\left({ℑ}_{h}^{3}(z_{j})-{\bar{ℑ}}_{h}^{3}\right)}^{2}}\sqrt{\sum_{j=1}^{N}{\left({ℜ}_{h}^{3}(z_{j})-{\bar{ℜ}}_{h}^{3}\right)}^{2}}} \end{array}\}.$$

The means, $\bar{ℑ}$ and $\bar{ℜ}$ are as defined in (10). This method (15) is not dependable whenever each element of *Z* in the FFSs has the same MD and NMD, respectively.

Finally, Amman et al. [68] presented a Spearman-like FFCCM as seen in (17):

(17) $${ϒ}_{7}(ℑ,ℜ)=\frac{\left(\rho_{m}(ℑ,ℜ)+\rho_{n}(ℑ,ℜ)+\rho_{h}(ℑ,ℜ)\right)}{3},$$

where $\rho_{m}(ℑ,ℜ)$, $\rho_{n}(ℑ,ℜ)$, and $\rho_{h}(ℑ,ℜ)$ are the Spearman rank correlation coefficients between ℑ and ℜ, and are defined in (18):

(18) $$\begin{array}{c} \rho_{m}(ℑ,ℜ)=1-\frac{6\sum_{j=1}^{N}d_{mj}^{2}}{\left(N^{3}-N\right)}\\ \rho_{n}(ℑ,ℜ)=1-\frac{6\sum_{j=1}^{N}d_{nj}^{2}}{\left(N^{3}-N\right)}\\ \rho_{h}(ℑ,ℜ)=1-\frac{6\sum_{j=1}^{N}d_{hj}^{2}}{\left(N^{3}-N\right)} \end{array}\},$$

where for j=1,2,⋯,N, $d_{mj}=R({ℑ}_{m}(z_{j}))-R({ℜ}_{m}(z_{j}))$ are the differences in the ranks of the MDs, $d_{nj}=R({ℑ}_{n}(z_{j}))-R({ℜ}_{n}(z_{j}))$ are the differences in the ranks of the NMDs, and $d_{hj}=R({ℑ}_{h}(z_{j}))-R({ℜ}_{h}(z_{j}))$ are the differences in the ranks of the PFHMs.

The method (17) does not incorporate the properties of FFSs and it uses crisp Spearman ranks. In addition, the method becomes unreliable whenever |Z|=1.

## 3 New FFCCM

Owing to the drawbacks in the existing FFCCMs in terms of precision and inability to measure FFCC in some cases, we construct a new FFCCM by replacing the crisp rank differences in [68] with the taxicab differences of the Fermatean fuzzy parameters. The new FFCCM is presented in the following definition:

Definition 4 Suppose ℑ and ℜ are FFSs in $Z=\{z_{1},z_{2},\cdots ,z_{N}\}$, $N\in Z^{+}$. Then, the new FFCCM is defined as (19):

(19) $${ϒ}_{\sigma }(ℑ,ℜ)=\frac{\left(\rho_{\sigma m}(ℑ,ℜ)+\rho_{\sigma n}(ℑ,ℜ)+\rho_{\sigma h}(ℑ,ℜ)\right)}{3},$$

where the weighted Spearman rank correlation coefficients between ℑ and ℜ are defined in (20):

(20) $$\begin{array}{c} \rho_{\sigma m}(ℑ,ℜ)=1-\frac{6\sum_{j=1}^{N}\sigma_{j}{\left[\max\{{ℑ}_{m}^{3}(z_{j}),{ℜ}_{m}^{3}(z_{j})\}-\min\{{ℑ}_{m}^{3}(z_{j}),{ℜ}_{m}^{3}(z_{j})\}\right]}^{3}}{{\left(N+1\right)}^{3}-(N+1)}\\ \rho_{\sigma n}(ℑ,ℜ)=1-\frac{6\sum_{j=1}^{N}\sigma_{j}{\left[\max\{{ℑ}_{n}^{3}(z_{j}),{ℜ}_{n}^{3}(z_{j})\}-\min\{{ℑ}_{n}^{3}(z_{j}),{ℜ}_{n}^{3}(z_{j})\}\right]}^{3}}{{\left(N+1\right)}^{3}-(N+1)}\\ \rho_{\sigma h}(ℑ,ℜ)=1-\frac{6\sum_{j=1}^{N}\sigma_{j}{\left[\max\{{ℑ}_{h}^{3}(z_{j}),{ℜ}_{h}^{3}(z_{j})\}-\min\{{ℑ}_{h}^{3}(z_{j}),{ℜ}_{h}^{3}(z_{j})\}\right]}^{3}}{{\left(N+1\right)}^{3}-(N+1)} \end{array}\}.$$

The weights $\sigma_{j}$ of $z_{j}\in Z$ are defined in (21):

(21) $$\sigma_{j}=\frac{\frac{3({ℑ}_{m}^{3}(z_{j})+{ℜ}_{m}^{3}(z_{j}))+({ℑ}_{n}^{3}(z_{j})+{ℜ}_{n}^{3}(z_{j}))+({ℑ}_{h}^{3}(z_{j})+{ℜ}_{h}^{3}(z_{j}))}{3}}{\sum_{j=1}^{N}(\frac{3({ℑ}_{m}^{3}(z_{j})+{ℜ}_{m}^{3}(z_{j}))+({ℑ}_{n}^{3}(z_{j})+{ℜ}_{n}^{3}(z_{j}))+({ℑ}_{h}^{3}(z_{j})+{ℜ}_{h}^{3}(z_{j}))}{3})},$$

where $\sum_{j=1}^{N}\sigma_{j}=1\;\text{and}\;\sigma_{j}\in [0,1]$.

Without considering the effects of the weights $\sigma_{j}$ of $z_{j}\in Z$, we get (22):

(22) $$ϒ(ℑ,ℜ)=\frac{\left(\rho_{m}(ℑ,ℜ)+\rho_{n}(ℑ,ℜ)+\rho_{h}(ℑ,ℜ)\right)}{3},$$

where the Spearman rank correlation coefficients between ℑ and ℜ are defined in (23):

(23) $$\begin{array}{c} \rho_{m}(ℑ,ℜ)=1-\frac{6\sum_{j=1}^{N}{\left[\max\{{ℑ}_{m}^{3}(z_{j}),{ℜ}_{m}^{3}(z_{j})\}-\min\{{ℑ}_{m}^{3}(z_{j}),{ℜ}_{m}^{3}(z_{j})\}\right]}^{3}}{{\left(N+1\right)}^{3}-(N+1)}\\ \rho_{n}(ℑ,ℜ)=1-\frac{6\sum_{j=1}^{N}{\left[\max\{{ℑ}_{n}^{3}(z_{j}),{ℜ}_{n}^{3}(z_{j})\}-\min\{{ℑ}_{n}^{3}(z_{j}),{ℜ}_{n}^{3}(z_{j})\}\right]}^{3}}{{\left(N+1\right)}^{3}-(N+1)}\\ \rho_{h}(ℑ,ℜ)=1-\frac{6\sum_{j=1}^{N}{\left[\max\{{ℑ}_{h}^{3}(z_{j}),{ℜ}_{h}^{3}(z_{j})\}-\min\{{ℑ}_{h}^{3}(z_{j}),{ℜ}_{h}^{3}(z_{j})\}\right]}^{3}}{{\left(N+1\right)}^{3}-(N+1)} \end{array}\}.$$

Next, we theoretically show that the new FFCCM fulfills the axioms of FFCC.

Theorem 1 *The new FFCCM,*ϒ(ℑ,ℜ)*between FFSs* ℑ *and* ℜ *in Z fulfills the conditions of FFCC.*

See the Appendix for the proof of Theorem 1.

Theorem 2 *The new weighted FFCCM,*${ϒ}_{\sigma }(ℑ,ℜ)$*between FFSs* ℑ *and* ℜ *in Z satisfies the conditions of FFCC.*

See the Appendix for the proof of Theorem 2.

### 3.1 Analysis of the FFCCMs

Some computation illustrations of the existing FFCCMs and the new FFCCM are discussed to pinpoint the drawbacks of the existing methods. To see the limitation of Kirisci's [64] method, the following example is considered.

Example 3.1 Suppose $ℑ=\{(z_{1},\frac{1}{2},0),(z_{2},0,\frac{3}{5})\}$ and $ℜ=\{(z_{1},0,1),(z_{2},1,0)\}$ are FFSs defined in $Z=\{z_{1},z_{2}\}$. By using (4) and (5), we have ρ(ℑ,ℜ)=0, ρ(ℑ,ℑ)=1.8308, ρ(ℜ,ℜ)=2 and thus, ${ϒ}_{1}(ℑ,ℜ)=\frac{0}{\sqrt{1.8308\times 2}}={ϒ}_{2}(ℑ,ℜ)=\frac{0}{\max\{1.8308,2\}}=0$. The result shows there is no correlation between ℑ and ℜ, which is not so. The correlation coefficient value is zero because of the alternating zero MDs and NMDs. In consequence, this analysis cannot be dependable. The drawback of these FFCCMs is that, they cannot estimate the FFCC with interchanging zero MDs and NMDs, respectively. On the other hand, the new FFCCM yields ϒ(ℑ,ℜ)=0.7363, which shows a positive correlation exists between the FFSs. While the new FFCCM is able to measure the correlation coefficient between the FFSs, ${ϒ}_{1}$ and ${ϒ}_{2}$ are unable to measure the FFCC.

For Bhatia et al.'s [65] method, we present the following examples:

Example 3.2 Suppose $ℑ=\{(z_{1},1,0)\}$ and $ℜ=\{(z_{1},0,1)\}$ are FFSs defined in $Z=\{z_{1}\}$. By using (7), we have ${ϒ}_{3}(ℑ,ℜ)=\frac{\infty }{\sqrt{\infty \times \infty }}=\frac{\infty }{\infty }$ since ρ(ℑ,ℜ)=ρ(ℑ,ℑ)=ρ(ℜ,ℜ)=∞ and N=1. The drawback of this FFCCM is that, it yields value that is not defined in [−1,1] or [0,1] and so violates C1. But, the new FFCCM yields ϒ(ℑ,ℜ)=0.3333, which shows correlation exists between the FFSs.

Example 3.3 If $ℑ=\{(z_{1},\frac{1}{4},\frac{1}{8}),(z_{2},\frac{1}{4},\frac{1}{8})\}$ and $ℜ=\{(z_{1},\frac{1}{2},\frac{1}{4}),(z_{2},\frac{1}{2},\frac{1}{4})\}$ are FFSs defined in $Z=\{z_{1},z_{2}\}$. By using (7), we have the following values:

$${\bar{ℑ}}_{m}=\frac{1}{4},{\bar{ℑ}}_{n}=\frac{1}{8},{\bar{ℑ}}_{h}=0.9941,{\bar{ℜ}}_{m}=\frac{1}{2},{\bar{ℜ}}_{n}=\frac{1}{4},{\bar{ℜ}}_{h}=0.9507,d(ℑ)=d(ℜ)=0,\rho (ℑ,ℜ)=\rho (ℑ,ℑ)=\rho (ℜ,ℜ)=0.$$

Thus, ${ϒ}_{3}(ℑ,ℜ)=\frac{0}{\sqrt{0\times 0}}=\frac{0}{0}$, which violates C1 of the FFCC conditions. On the other hand, the new FFCCM gives ϒ(ℑ,ℜ)=0.9995, which indicates a strong correlation coefficient exists between the FFSs.

Though one of the approaches in [66] is reliable in terms of efficiency and mathematical correctness, it losses accuracy as *N* increases. This can be verified as follows:

Example 3.4 Suppose we have two FFSs defined in $Z=\{z_{1},z_{2},z_{3},z_{4},z_{5},z_{6}\}$ as

$$ℑ=\{(z_{1},\frac{1}{4},\frac{1}{8}),(z_{2},\frac{1}{4},\frac{1}{8}),(z_{3},\frac{1}{2},\frac{1}{4}),(z_{4},\frac{1}{8},\frac{1}{2}),(z_{5},\frac{3}{10},\frac{7}{10}),(z_{6},\frac{1}{2},\frac{3}{5})\}\;\text{and}ℜ=\{(z_{1},\frac{1}{2},\frac{1}{4}),(z_{2},\frac{1}{2},\frac{1}{4}),(z_{3},\frac{1}{2},\frac{1}{8}),(z_{4},\frac{2}{5},\frac{1}{8}),(z_{5},\frac{3}{5},\frac{1}{2}),(z_{6},\frac{7}{10},\frac{3}{10})\}.$$

By using (11), we have ${ϒ}_{4}(ℑ,ℜ)=0.9441$, which shows that ℑ and ℜ are well related. But, the new FFCCM gives ϒ(ℑ,ℜ)=0.9997, which is more precise than 0.9441. Although ${ϒ}_{4}$ yields a good result, the new FFCCM gives a better result by comparison.

The second method in [66] is not dependable and its limitation is similar to the FFCCMs in [64]. By applying it to Example 3.1, we get

$${ϒ}_{5}(ℑ,ℜ)=\frac{\sqrt[{3}]{0}}{\sqrt[{6}]{1.8308\times 2}}=0,$$

showing that no correlation exists between the FFSs. However, this information is not true. On the other hand, The new FFCCM yields ϒ(ℑ,ℜ)=0.7363, which shows a positive correlation exists between the FFSs. While the new FFCCM is able to measure the correlation coefficient between the FFSs, ${ϒ}_{5}$ is unable to measure the FFCC.

Gouli et al.'s [67] method is not dependable whenever each element of *Z* in the FFSs has the same MD and NMD. For instance, by using (15) to compute the FFCC between ℑ and ℜ in Example 3.3, we have ${ϒ}_{6}(ℑ,ℜ)=\frac{0}{0}$, which is not defined in [−1,1] or [0,1] and so violates C1. But, the new FFCCM yields ϒ(ℑ,ℜ)=0.9995, which is a strong correlation coefficient.

To see the limitation of Amman et al.'s [68] method, we consider the following example:

Example 3.5 If $ℑ=\{(z,\frac{1}{4},\frac{1}{2})\}$ and $ℜ=\{(z,\frac{1}{2},\frac{1}{8})\}$ are FFSs defined in Z={z}. By using (17), we have the following values:

$$d_{m1}^{2}=d_{n1}^{2}=d_{h1}^{2}=0,\rho_{m}(ℑ,ℜ)=\rho_{n}(ℑ,ℜ)=\rho_{h}(ℑ,ℜ)=\infty ,$$

and so ${ϒ}_{7}(ℑ,ℜ)=\infty$, which violates C1 of the FFCC conditions. On the contrary, the new FFCCM yields ϒ(ℑ,ℜ)=0.9989, which is a strong correlation coefficient.

From the considered examples, it is fit to say that the FFCCMs ${ϒ}_{1}$, ${ϒ}_{2}$, ${ϒ}_{3}$, ${ϒ}_{5}$, ${ϒ}_{6}$, and ${ϒ}_{7}$ in [64], [65], [66], [67], [68] are not suitable and reliable FFCCMs. Although ${ϒ}_{4}$ in [66] is suitable and appropriate measure, it lacks precision as *N* increases. On the contrary, the new FFCCM gives consistent and reliable results which satisfied the axioms of FFCC.

## 4 Insecurity assessment based on FFCCMs

The process of insecurity assessment is a herculean assignment because of the unpredictable nature of insecurity. Many studies have been carried out to identify the most insecure state in the NCRN. These studies yielded no result because they do not incorporate soft computing tool, which is able to tackle the uncertainty, unpredictability, and fuzziness of the insecurity situations in the region. Because of the efficiency of the FFSs in tackling uncertainty and fuzziness in decision-making, it is deployed to determine the most insecure state in the region. To achieve this, the knowledge-based method of data collection and FFCCMs-based TOPSIS technique are employed.

### 4.1 Experimental example

The study is carried out in the NCRN to assess the region's insecurity situations. The outcome of the assessment will enhance reliable travel advisory for both local and foreign travelers. The NCRN is comprised of six states alongside the FCT. The NCRN is an insecurity prone area with several indicators of insecurity. According to the investigations in [4], [5], [6], [8], the security concerns namely: struggles/mayhems due to mineral resources, ethno-religious conflicts/terrorism, porous borders, farmer-herder clashes, political agitations, banditry and kidnapping, and weak/complicit security architecture are the main causes of security crises in the NCRN. The indicators of insecurity are represented by a set, $Z=\{z_{1},z_{2},z_{3},z_{4},z_{5},z_{6},z_{7}\}$, where $z_{1}$ represents ethno-religious conflicts/terrorism, $z_{2}$ represents farmer-herder clashes due to open grazing practice (i.e., clashes owing to the use of land, water, and grazing routes), $z_{3}$ represents banditry and kidnapping due to poverty arises from pervasive material inequalities and corruption, $z_{4}$ represents struggles/mayhems due to mineral resources, $z_{5}$ represents weak/complicit security architecture, $z_{6}$ represents porous borders, and $z_{7}$ represents political agitations.

The NCRN consists of Benue State, Nassarawa State, Kogi State, Niger State, Plateau State, Kwara State, and the FCT. The NCRN is represented by a family of FFSs denoted by $ℜ=\{{ℜ}_{1},{ℜ}_{2},{ℜ}_{3},{ℜ}_{4},{ℜ}_{5},{ℜ}_{6},{ℜ}_{7}\}$, which are defined in terms of the insecurity indicators *Z*, where ${ℜ}_{1}$ represents Benue State, ${ℜ}_{2}$ represents Nassarawa State, ${ℜ}_{3}$ represents Kogi State, ${ℜ}_{4}$ represents Niger State, ${ℜ}_{5}$ represents Plateau State, ${ℜ}_{6}$ represents Kwara State, and ${ℜ}_{7}$ represents the FCT.

#### 4.1.1 Knowledge-based expert systems

Expert systems are a field of AI that aims to give computerized decision-making skills comparable to those of a human expert in a certain topic [73]. They are intended to solve complex issues using a system of rules or algorithms that resemble human thinking processes. A KBS is an essential aspect of knowledge representation in AI. KBS is a computerized technique that uses a centralized information repository to aid decision-making. It uses information from human experts and utilize it in making complex decisions, just like a team of human experts. KBS frequently use LVs, which are variables expressed in terms of linguistic notions rather than numerical values.

The data for the evaluation were collected using knowledge-based expert system, where the thoughts of three security experts are obtained in terms of LVs. These security experts are familiar with the security crises in the region. The LVs with respect to the security indicators are; high, very high, very-very high, extremely high, extremely low, very-very low, very low, low, medium low, medium, and medium high with associated Fermatean fuzzy numbers (FFNs). The LVs with their associated FFNs are captured in Table 1.

**Table 1:** LVs for insecurity evaluation.

Linguistic Variables	FFNs
EH	(1.0,0.0)
VVH	(0.9,0.1)
VH	(0.8,0.2)
H	(0.7,0.3)
MH	(0.6,0.4)
M	(0.5,0.5)
ML	(0.4,0.6)
L	(0.3,0.7)
VL	(0.2,0.8)
VVL	(0.1,0.9)
EL	(0.0,1.0)

Three security experts were consulted to assess the security situations in the NCRN and give their expert opinions by using the LVs in Table 1, and their opinions are presented in Table 2. Next, the LVs are converted to FFNs presented in Table 3.

**Table 2:** Security experts' opinions.

NCRN	*z*_1_	*z*_2_	*z*_3_	*z*_4_	*z*_5_	*z*_6_	*z*_7_
Security Expert I Linguistic Variables							

ℜ_1_	VVH	EH	MH	VL	MH	VVL	EL
ℜ_2_	VH	VVH	ML	MH	M	L	MH
ℜ_3_	VVH	VH	VH	MH	MH	L	H
ℜ_4_	EH	VVH	VH	VH	H	VVH	VL
ℜ_5_	VVH	VVH	MH	VH	VH	L	MH
ℜ_6_	VH	VH	H	M	M	VH	M
ℜ_7_	VH	M	VH	M	M	ML	VVL

Security Expert II Linguistic Variables							

ℜ_1_	VH	VVH	H	L	MH	VL	VL
ℜ_2_	VVH	VH	M	M	L	VL	M
ℜ_3_	VH	H	VH	H	MH	ML	H
ℜ_4_	VVH	VH	H	VH	MH	VH	VL
ℜ_5_	VH	VH	H	H	VH	L	M
ℜ_6_	VVH	VH	MH	M	MH	H	MH
ℜ_7_	VH	MH	H	M	L	ML	VL

Security Expert III Linguistic Variables							

ℜ_1_	VVH	VH	ML	ML	MH	L	L
ℜ_2_	VH	VVH	M	MH	ML	VL	MH
ℜ_3_	VVH	VH	H	VH	H	M	VH
ℜ_4_	VH	H	VH	H	MH	H	VL
ℜ_5_	H	MH	H	MH	H	ML	MH
ℜ_6_	VVH	VH	M	M	MH	MH	H
ℜ_7_	VH	H	MH	MH	ML	L	VL

**Table 3:** FFNs of security experts.

NCRN	*z*_1_	*z*_2_	*z*_3_	*z*_4_	*z*_5_	*z*_6_	*z*_7_
FFNs of Security Expert I							

ℜ_1_	(0.9,0.1)	(1.0,0.0)	(0.6,0.4)	(0.2,0.8)	(0.6,0.4)	(0.1,0.9)	(0.0,1.0)
ℜ_2_	(0.8,0.2)	(0.9,0.1)	(0.4,0.6)	(0.6,0.4)	(0.5,0.5)	(0.3,0.7)	(0.6,0.4)
ℜ_3_	(0.9,0.1)	(0.8,0.2)	(0.8,0.2)	(0.6,0.4)	(0.6,0.4)	(0.3,0.7)	(0.7,0.3)
ℜ_4_	(1.0,0.0)	(0.9,0.1)	(0.8,0.2)	(0.8,0.2)	(0.7,0.3)	(0.9,0.1)	(0.2,0.8)
ℜ_5_	(0.9,0.1)	(0.9,0.1)	(0.6,0.4)	(0.8,0.2)	(0.8,0.2)	(0.3,0.7)	(0.6,0.4)
ℜ_6_	(0.8,0.2)	(0.8,0.2)	(0.7,0.3)	(0.5,0.5)	(0.5,0.5)	(0.8,0.2)	(0.5,0.5)
ℜ_7_	(0.8,0.2)	(0.5,0.5)	(0.8,0.2)	(0.5,0.5)	(0.5,0.5)	(0.4,0.6)	(0.1,0.9)

FFNs of Security Expert II							

ℜ_1_	(0.8,0.2)	(0.9,0.1)	(0.7,0.3)	(0.3,0.7)	(0.6,0.4)	(0.2,0.8)	(0.2,0.8)
ℜ_2_	(0.9,0.1)	(0.8,0.2)	(0.5,0.5)	(0.5,0.5)	(0.3,0.7)	(0.2,0.8)	(0.5,0.5)
ℜ_3_	(0.8,0.2)	(0.7,0.3)	(0.8,0.2)	(0.7,0.3)	(0.6,0.4)	(0.4,0.6)	(0.7,0.3)
ℜ_4_	(0.9,0.1)	(0.8,0.2)	(0.7,0.3)	(0.8,0.2)	(0.6,0.4)	(0.8,0.2)	(0.2,0.8)
ℜ_5_	(0.8,0.2)	(0.8,0.2)	(0.7,0.3)	(0.7,0.3)	(0.8,0.2)	(0.3,0.7)	(0.5,0.5)
ℜ_6_	(0.9,0.1)	(0.8,0.2)	(0.6,0.4)	(0.5,0.5)	(0.6,0.4)	(0.7,0.3)	(0.6,0.4)
ℜ_7_	(0.8,0.2)	(0.6,0.4)	(0.7,0.3)	(0.5,0.5)	(0.3,0.7)	(0.4,0.6)	(0.2,0.8)

FFNs of Security Expert III							

ℜ_1_	(0.9,0.1)	(0.8,0.2)	(0.4,0.6)	(0.4,0.6)	(0.6,0.4)	(0.3,0.7)	(0.3,0.7)
ℜ_2_	(0.8,0.2)	(0.9,0.1)	(0.5,0.5)	(0.6,0.4)	(0.4,0.6)	(0.2,0.8)	(0.6,0.4)
ℜ_3_	(0.9,0.1)	(0.8,0.2)	(0.7,0.3)	(0.8,0.2)	(0.7,0.3)	(0.5,0.5)	(0.8,0.2)
ℜ_4_	(0.8,0.2)	(0.7,0.3)	(0.8,0.2)	(0.7,0.3)	(0.6,0.4)	(0.7,0.3)	(0.2,0.8)
ℜ_5_	(0.7,0.3)	(0.6,0.4)	(0.7,0.3)	(0.6,0.4)	(0.7,0.3)	(0.4,0.6)	(0.6,0.4)
ℜ_6_	(0.9,0.1)	(0.8,0.2)	(0.5,0.5)	(0.5,0.5)	(0.6,0.4)	(0.6,0.4)	(0.7,0.3)
ℜ_7_	(0.8,0.2)	(0.7,0.3)	(0.6,0.4)	(0.6,0.4)	(0.4,0.6)	(0.3,0.7)	(0.2,0.8)

Subsequently, the TOPSIS algorithm for the security assessment process is presented. The first algorithm is without the weights of $z_{j}$ and the second algorithm incorporates the weights of $z_{j}$.

#### 4.1.2 TOPSIS algorithm without WNFFDM

**Step 1:** Find the FFDM, ${\overset{˜}{ℜ}}_{i}={\left\{z_{j}({ℜ}_{i})\right\}}_{\left(K\times N\right)}$ for i=1,2,⋯,K, j=1,2,⋯,N, where ${ℜ}_{i}$ and $z_{j}$ represent the states of the NCRN and insecurity indicators, respectively.

**Step 2:** Determine the CC and BC. The CC is the least $z_{j}$ and BC are the non-least $z_{j}$.

**Step 3:** Find the NFFDM represented by (24):

(24) $${\overset{˜}{ℜ}}_{i}^{⁎}={\left({ℜ}_{i_{m}}(z_{j}),{ℜ}_{i_{n}}(z_{j})\right)}_{K\times N},$$

where $\left({ℜ}_{i_{m}}(z_{j}),{ℜ}_{i_{n}}(z_{j})\right)$ are the FFNs, and ${\overset{˜}{ℜ}}_{i}^{⁎}$ is defined by (25):

(25) $${\overset{˜}{ℜ}}_{i}^{⁎}=\left\{ \begin{array}{cc} ({ℜ}_{i_{m}}(z_{j}),{ℜ}_{i_{n}}(z_{j}))\; & \;\text{for BC of}\;{ℜ}_{i}\\ ({ℜ}_{i_{n}}(z_{j}),{ℜ}_{i_{m}}(z_{j}))\; & \;\text{for CC of}\;{ℜ}_{i} \end{array}\right.$$

**Step 4:** Obtain the PIS, ${\overset{˜}{ℜ}}^{⁎+}=\{{\overset{˜}{ℜ}}_{1}^{⁎+},{\overset{˜}{ℜ}}_{2}^{⁎+},\cdots ,{\overset{˜}{ℜ}}_{N}^{⁎+}\}$ and the NIS, ${\overset{˜}{ℜ}}^{⁎-}=\{{\overset{˜}{ℜ}}_{1}^{⁎-},{\overset{˜}{ℜ}}_{2}^{⁎-},\cdots ,{\overset{˜}{ℜ}}_{N}^{⁎-}\}$, which are defined by (26):

(26) $$\begin{array}{c} {\overset{˜}{ℜ}}^{⁎+}=\left\{ \begin{array}{cc} (\max\{{\overset{˜}{ℜ}}_{m_{i}}^{⁎}(z_{j})\},\min\{{\overset{˜}{ℜ}}_{n_{i}}^{⁎}(z_{j})\}),\; & \;\text{if}\;z_{j}\;\text{is the BC}\\ (\min\{{\overset{˜}{ℜ}}_{m_{i}}^{⁎}(z_{j})\},\max\{{\overset{˜}{ℜ}}_{n_{i}}^{⁎}(z_{j})\}),\; & \;\text{if}\;z_{j}\;\text{is the CC} \end{array}\right.\\ {\overset{˜}{ℜ}}^{⁎-}=\left\{ \begin{array}{cc} (\min\{{\overset{˜}{ℜ}}_{m_{i}}^{⁎}(z_{j})\},\max\{{\overset{˜}{ℜ}}_{n_{i}}^{⁎}(z_{j})\}),\; & \;\text{if}\;z_{j}\;\text{is the BC}\\ (\max\{{\overset{˜}{ℜ}}_{m_{i}}^{⁎}(z_{j})\},\min\{{\overset{˜}{ℜ}}_{n_{i}}^{⁎}(z_{j})\}),\; & \;\text{if}\;z_{j}\;\text{is the CC} \end{array}\right. \end{array}\}.$$

**Step 5:** Obtain the correlation coefficients between ${\overset{˜}{ℜ}}^{⁎-}$ and ${ℜ}_{i}$, and ${\overset{˜}{ℜ}}^{⁎+}$ and ${ℜ}_{i}$ by using the FFCCMs for i=1,2,⋯,K.

**Step 6:** Find the closeness coefficients, $\Theta_{i}({ℜ}_{i})$ by using (27):

(27) $$\Theta_{i}({ℜ}_{i})=\frac{\Theta_{i}^{+}({ℜ}_{i})}{\Theta_{i}^{+}({ℜ}_{i})+\Theta_{i}^{-}({ℜ}_{i})},$$

for i=1,2,⋯,K, where $\Theta_{i}^{+}({ℜ}_{i})=ϒ({\overset{˜}{ℜ}}_{i}^{⁎+},{ℜ}_{i})$ and $\Theta_{i}^{-}({ℜ}_{i})=ϒ({\overset{˜}{ℜ}}_{i}^{⁎-},{ℜ}_{i})$. In case $ϒ({\overset{˜}{ℜ}}_{i}^{⁎-},{ℜ}_{i}),ϒ({\overset{˜}{ℜ}}_{i}^{⁎+},{ℜ}_{i})\in [-1,1]$, we find $\Theta_{i}({ℜ}_{i})$ by (28):

(28) $$\begin{array}{c} \Theta_{i}^{+}({ℜ}_{i})=\frac{ϒ({\overset{˜}{ℜ}}_{i}^{⁎+},{ℜ}_{i})-{ϒ}_{\min}({\overset{˜}{ℜ}}_{i}^{⁎+},{ℜ}_{i})}{{ϒ}_{\max}({\overset{˜}{ℜ}}_{i}^{⁎+},{ℜ}_{i})-{ϒ}_{\min}({\overset{˜}{ℜ}}_{i}^{⁎+},{ℜ}_{i})}\\ \Theta_{i}^{-}({ℜ}_{i})=\frac{ϒ({\overset{˜}{ℜ}}_{i}^{⁎-},{ℜ}_{i})-{ϒ}_{\min}({\overset{˜}{ℜ}}_{i}^{⁎-},{ℜ}_{i})}{{ϒ}_{\max}({\overset{˜}{ℜ}}_{i}^{⁎-},{ℜ}_{i})-{ϒ}_{\min}({\overset{˜}{ℜ}}_{i}^{⁎-},{ℜ}_{i})} \end{array}\}.$$

**Step 7:** Select the state with the greatest closeness coefficient as the most insecure state.

**Figure 1:**
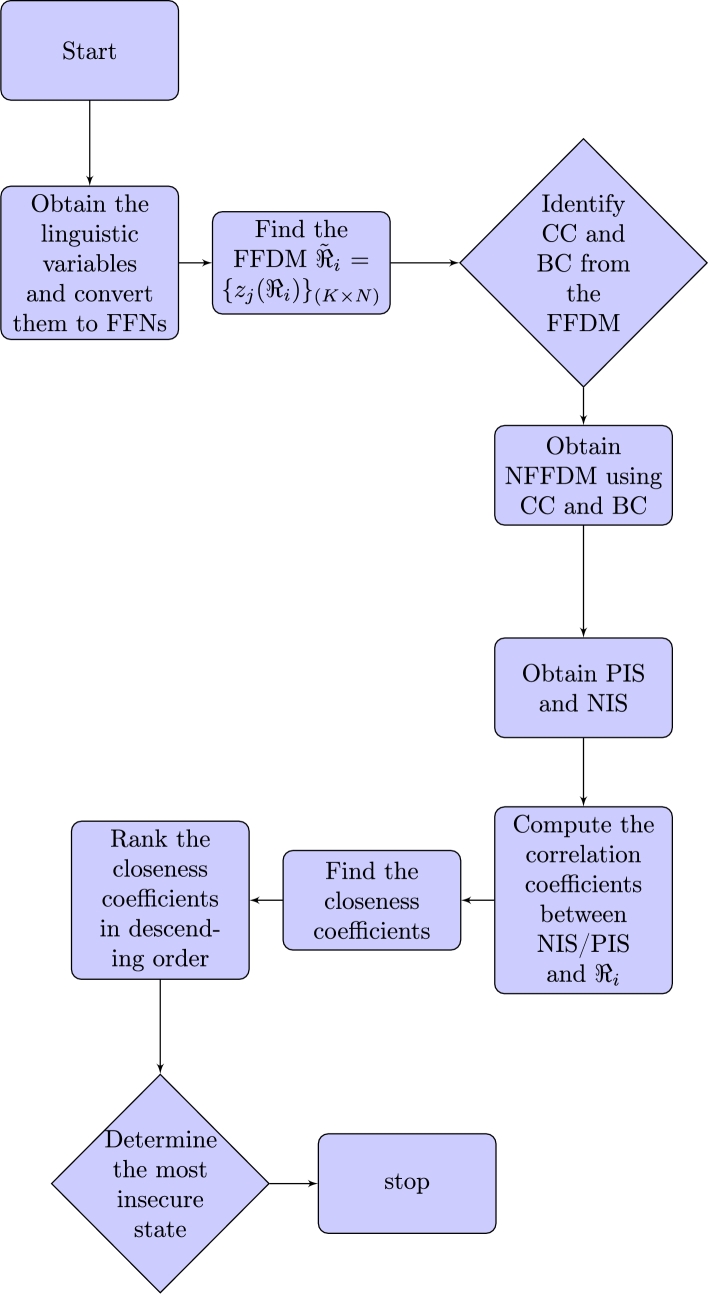
Flowchart of algorithm without weights.

The flowchart of the algorithm without weights is shown in Fig. 1.

#### 4.1.3 TOPSIS algorithm with WNFFDM

**Step 1:** Same as Step 1 of the algorithm without weights.

**Step 2:** Find the weights of $z_{j}$ by using (21).

**Step 3:** Same as Step 2 of the algorithm without weights.

**Step 4:** Same as Step 3 of the algorithm without weights.**Step 5:** Find the WNFFDM, $\sigma_{j}{\overset{˜}{ℜ}}_{i}^{⁎}$ using (29):

(29) $$\sigma_{j}{\overset{˜}{ℜ}}_{i}^{⁎}=(1-{\left(1-{ℜ}_{i_{m}}^{3}(z_{j})\right)}^{\sigma_{j}},{\left({ℜ}_{i_{n}}^{3}(z_{j})\right)}^{\sigma_{j}}).$$

**Step 6:** Obtain the weighted PIS, $\sigma {\overset{˜}{ℜ}}^{⁎+}=\{\sigma {\overset{˜}{ℜ}}_{1}^{⁎+},\cdots ,\sigma {\overset{˜}{ℜ}}_{N}^{⁎+}\}$ and the weighted NIS, $\sigma {\overset{˜}{ℜ}}^{⁎-}=\{\sigma {\overset{˜}{ℜ}}_{1}^{⁎-},\cdots ,\sigma {\overset{˜}{ℜ}}_{N}^{⁎-}\}$, which are defined by (30):

(30) $$\begin{array}{c} \sigma {\overset{˜}{ℜ}}^{⁎+}=\left\{ \begin{array}{cc} (\max\{\sigma_{j}{\overset{˜}{ℜ}}_{m_{i}}^{⁎}(z_{j})\},\min\{\sigma_{j}{\overset{˜}{ℜ}}_{n_{i}}^{⁎}(z_{j})\}),\; & \;\text{if}\;z_{j}\;\text{is the BC}\\ (\min\{\sigma_{j}{\overset{˜}{ℜ}}_{m_{i}}^{⁎}(z_{j})\},\max\{\sigma_{j}{\overset{˜}{ℜ}}_{n_{i}}^{⁎}(z_{j})\}),\; & \;\text{if}\;z_{j}\;\text{is the CC} \end{array}\right.\\ \sigma {\overset{˜}{ℜ}}^{⁎-}=\left\{ \begin{array}{cc} (\min\{\sigma_{j}{\overset{˜}{ℜ}}_{m_{i}}^{⁎}(z_{j})\},\max\{\sigma_{j}{\overset{˜}{ℜ}}_{n_{i}}^{⁎}(z_{j})\}),\; & \;\text{if}\;z_{j}\;\text{is the BC}\\ (\max\{\sigma_{j}{\overset{˜}{ℜ}}_{m_{i}}^{⁎}(z_{j})\},\min\{\sigma_{j}{\overset{˜}{ℜ}}_{n_{i}}^{⁎}(z_{j})\}),\; & \;\text{if}\;z_{j}\;\text{is the CC} \end{array}\right. \end{array}\}.$$

**Step 7:** Obtain the correlation coefficients between $\sigma {\overset{˜}{ℜ}}^{⁎-}$ and ${ℜ}_{i}$, and $\sigma {\overset{˜}{ℜ}}^{⁎+}$ and ${ℜ}_{i}$ by using the FFCCMs for i=1,2,⋯,K.

**Step 8:** Find the closeness coefficients, $\sigma \Theta_{i}({ℜ}_{i})$ by using (31):

(31) $$\sigma \Theta_{i}({ℜ}_{i})=\frac{\sigma \Theta_{i}^{+}({ℜ}_{i})}{\sigma \Theta_{i}^{+}({ℜ}_{i})+\sigma \Theta_{i}^{-}({ℜ}_{i})},$$

for i=1,2,⋯,K, where $\sigma \Theta_{i}^{+}({ℜ}_{i})=ϒ(\sigma {\overset{˜}{ℜ}}_{i}^{⁎+},{ℜ}_{i})$ and $\sigma \Theta_{i}^{-}({ℜ}_{i})=ϒ(\sigma {\overset{˜}{ℜ}}_{i}^{⁎-},{ℜ}_{i})$. In case $ϒ(\sigma {\overset{˜}{ℜ}}_{i}^{⁎-},{ℜ}_{i}),ϒ(\sigma {\overset{˜}{ℜ}}_{i}^{⁎+},{ℜ}_{i})\in [-1,1]$, we find $\sigma \Theta_{i}^{+}({ℜ}_{i})$ and $\sigma \Theta_{i}^{-}({ℜ}_{i})$ by (32):

(32) $$\begin{array}{c} \sigma \Theta_{i}^{+}({ℜ}_{i})=\frac{ϒ(\sigma {\overset{˜}{ℜ}}_{i}^{⁎+},{ℜ}_{i})-{ϒ}_{\min}(\sigma {\overset{˜}{ℜ}}_{i}^{⁎+},{ℜ}_{i})}{{ϒ}_{\max}(\sigma {\overset{˜}{ℜ}}_{i}^{⁎+},{ℜ}_{i})-{ϒ}_{\min}(\sigma {\overset{˜}{ℜ}}_{i}^{⁎+},{ℜ}_{i})}\\ \sigma \Theta_{i}^{-}({ℜ}_{i})=\frac{ϒ(\sigma {\overset{˜}{ℜ}}_{i}^{⁎-},{ℜ}_{i})-{ϒ}_{\min}(\sigma {\overset{˜}{ℜ}}_{i}^{⁎-},{ℜ}_{i})}{{ϒ}_{\max}(\sigma {\overset{˜}{ℜ}}_{i}^{⁎-},{ℜ}_{i})-{ϒ}_{\min}(\sigma {\overset{˜}{ℜ}}_{i}^{⁎-},{ℜ}_{i})} \end{array}\}.$$

**Step 9:** Same as Step 7 of the algorithm without weights.

**Figure 2:**
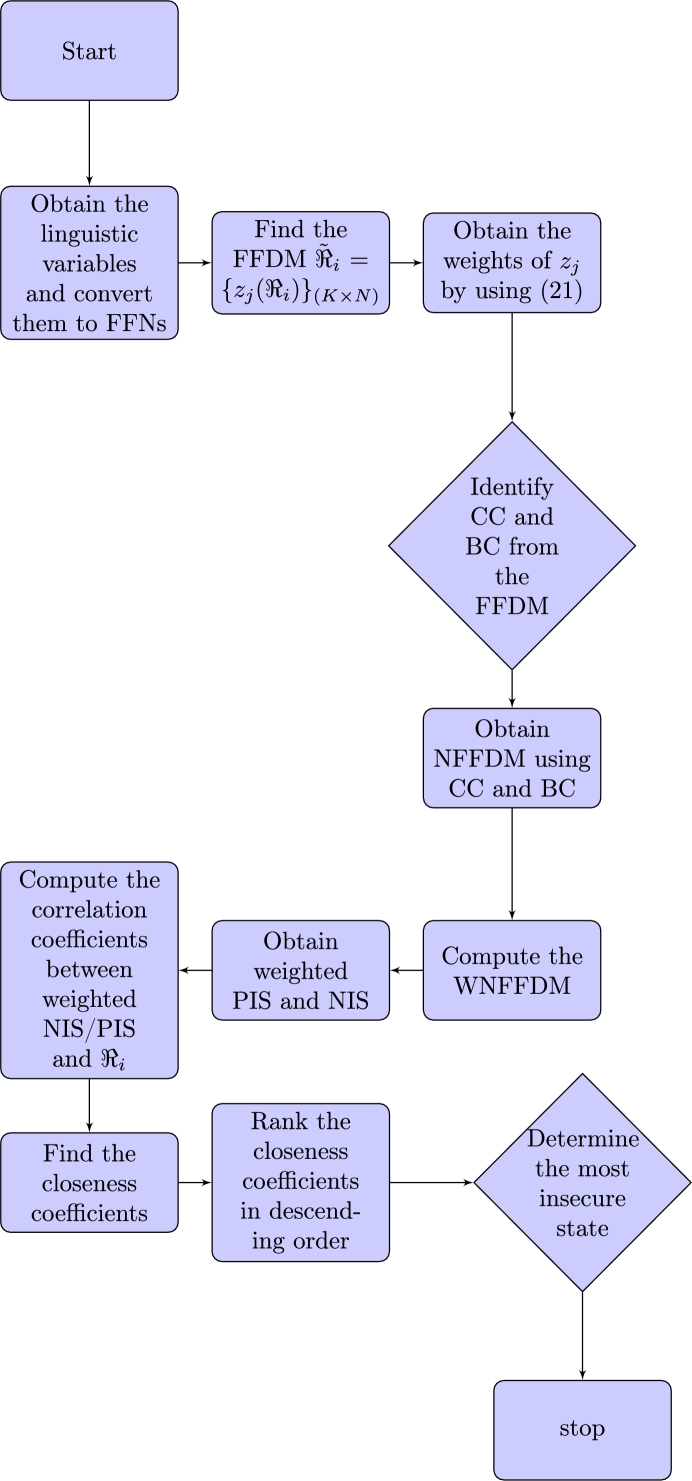
Flowchart of algorithm with weights.

The flowchart of the algorithm without weights is shown in Fig. 2.

#### 4.1.4 Implementation

The two algorithms are implemented side-by-side. For ease of computation, the averages of the FFNs in Table 3 are computed and presented in Table 4.

**Table 4:** FFDM for the security experts.

NCRN	*z*_1_	*z*_2_	*z*_3_	*z*_4_	*z*_5_	*z*_6_	*z*_7_
ℜ_1_	$\left(\begin{array}{c} 0.8667\\ 0.1333 \end{array}\right)$	$\left(\begin{array}{c} 0.9000\\ 0.1000 \end{array}\right)$	$\left(\begin{array}{c} 0.5667\\ 0.4333 \end{array}\right)$	$\left(\begin{array}{c} 0.3000\\ 0.7000 \end{array}\right)$	$\left(\begin{array}{c} 0.6000\\ 0.4000 \end{array}\right)$	$\left(\begin{array}{c} 0.2000\\ 0.8000 \end{array}\right)$	$\left(\begin{array}{c} 0.1667\\ 0.8333 \end{array}\right)$
ℜ_2_	$\left(\begin{array}{c} 0.8333\\ 0.1667 \end{array}\right)$	$\left(\begin{array}{c} 0.8667\\ 0.1333 \end{array}\right)$	$\left(\begin{array}{c} 0.4667\\ 0.5333 \end{array}\right)$	$\left(\begin{array}{c} 0.5667\\ 0.4333 \end{array}\right)$	$\left(\begin{array}{c} 0.4000\\ 0.6000 \end{array}\right)$	$\left(\begin{array}{c} 0.2333\\ 0.7667 \end{array}\right)$	$\left(\begin{array}{c} 0.5667\\ 0.4333 \end{array}\right)$
ℜ_3_	$\left(\begin{array}{c} 0.8667\\ 0.1333 \end{array}\right)$	$\left(\begin{array}{c} 0.7667\\ 0.2333 \end{array}\right)$	$\left(\begin{array}{c} 0.7667\\ 0.2333 \end{array}\right)$	$\left(\begin{array}{c} 0.7000\\ 0.3000 \end{array}\right)$	$\left(\begin{array}{c} 0.6333\\ 0.3667 \end{array}\right)$	$\left(\begin{array}{c} 0.4000\\ 0.6000 \end{array}\right)$	$\left(\begin{array}{c} 0.7333\\ 0.2667 \end{array}\right)$
ℜ_4_	$\left(\begin{array}{c} 0.9000\\ 0.1000 \end{array}\right)$	$\left(\begin{array}{c} 0.8000\\ 0.2000 \end{array}\right)$	$\left(\begin{array}{c} 0.7667\\ 0.2333 \end{array}\right)$	$\left(\begin{array}{c} 0.7667\\ 0.2333 \end{array}\right)$	$\left(\begin{array}{c} 0.6333\\ 0.3667 \end{array}\right)$	$\left(\begin{array}{c} 0.8000\\ 0.2000 \end{array}\right)$	$\left(\begin{array}{c} 0.2000\\ 0.8000 \end{array}\right)$
ℜ_5_	$\left(\begin{array}{c} 0.8000\\ 0.2000 \end{array}\right)$	$\left(\begin{array}{c} 0.7667\\ 0.2333 \end{array}\right)$	$\left(\begin{array}{c} 0.6667\\ 0.3333 \end{array}\right)$	$\left(\begin{array}{c} 0.7000\\ 0.3000 \end{array}\right)$	$\left(\begin{array}{c} 0.7667\\ 0.2333 \end{array}\right)$	$\left(\begin{array}{c} 0.3333\\ 0.6667 \end{array}\right)$	$\left(\begin{array}{c} 0.5667\\ 0.3333 \end{array}\right)$
ℜ_6_	$\left(\begin{array}{c} 0.8667\\ 0.1333 \end{array}\right)$	$\left(\begin{array}{c} 0.8000\\ 0.2000 \end{array}\right)$	$\left(\begin{array}{c} 0.6000\\ 0.4000 \end{array}\right)$	$\left(\begin{array}{c} 0.5000\\ 0.5000 \end{array}\right)$	$\left(\begin{array}{c} 0.5667\\ 0.4333 \end{array}\right)$	$\left(\begin{array}{c} 0.7000\\ 0.3000 \end{array}\right)$	$\left(\begin{array}{c} 0.6000\\ 0.4000 \end{array}\right)$
ℜ_7_	$\left(\begin{array}{c} 0.8000\\ 0.2000 \end{array}\right)$	$\left(\begin{array}{c} 0.6000\\ 0.4000 \end{array}\right)$	$\left(\begin{array}{c} 0.7000\\ 0.3000 \end{array}\right)$	$\left(\begin{array}{c} 0.5333\\ 0.4667 \end{array}\right)$	$\left(\begin{array}{c} 0.4000\\ 0.6000 \end{array}\right)$	$\left(\begin{array}{c} 0.3667\\ 0.6333 \end{array}\right)$	$\left(\begin{array}{c} 0.1667\\ 0.8333 \end{array}\right)$

A careful study of the insecurity indicators shows that political agitations are the least cause of insecurity in the NCRN. Thus, the CC is $z_{7}$. From Table 4, we obtained the weights of the insecurity indicators as follows:

σ={0.1966,0.1773,0.1399,0.1294,0.1289,0.1143,0.1134}.

By using Table 4, the NFFDM in Table 5 is obtained.

**Table 5:** NFFDM for the security experts.

NCRN	*z*_1_	*z*_2_	*z*_3_	*z*_4_	*z*_5_	*z*_6_	*z*_7_
ℜ_1_	$\left(\begin{array}{c} 0.8667\\ 0.1333 \end{array}\right)$	$\left(\begin{array}{c} 0.9000\\ 0.1000 \end{array}\right)$	$\left(\begin{array}{c} 0.5667\\ 0.4333 \end{array}\right)$	$\left(\begin{array}{c} 0.3000\\ 0.7000 \end{array}\right)$	$\left(\begin{array}{c} 0.6000\\ 0.4000 \end{array}\right)$	$\left(\begin{array}{c} 0.2000\\ 0.8000 \end{array}\right)$	$\left(\begin{array}{c} 0.8333\\ 0.1667 \end{array}\right)$
ℜ_2_	$\left(\begin{array}{c} 0.8333\\ 0.1667 \end{array}\right)$	$\left(\begin{array}{c} 0.8667\\ 0.1333 \end{array}\right)$	$\left(\begin{array}{c} 0.4667\\ 0.5333 \end{array}\right)$	$\left(\begin{array}{c} 0.5667\\ 0.4333 \end{array}\right)$	$\left(\begin{array}{c} 0.4000\\ 0.6000 \end{array}\right)$	$\left(\begin{array}{c} 0.2333\\ 0.7667 \end{array}\right)$	$\left(\begin{array}{c} 0.4333\\ 0.5667 \end{array}\right)$
ℜ_3_	$\left(\begin{array}{c} 0.8667\\ 0.1333 \end{array}\right)$	$\left(\begin{array}{c} 0.7667\\ 0.2333 \end{array}\right)$	$\left(\begin{array}{c} 0.7667\\ 0.2333 \end{array}\right)$	$\left(\begin{array}{c} 0.7000\\ 0.3000 \end{array}\right)$	$\left(\begin{array}{c} 0.6333\\ 0.3667 \end{array}\right)$	$\left(\begin{array}{c} 0.4000\\ 0.6000 \end{array}\right)$	$\left(\begin{array}{c} 0.2667\\ 0.7333 \end{array}\right)$
ℜ_4_	$\left(\begin{array}{c} 0.9000\\ 0.1000 \end{array}\right)$	$\left(\begin{array}{c} 0.8000\\ 0.2000 \end{array}\right)$	$\left(\begin{array}{c} 0.7667\\ 0.2333 \end{array}\right)$	$\left(\begin{array}{c} 0.7667\\ 0.2333 \end{array}\right)$	$\left(\begin{array}{c} 0.6333\\ 0.3667 \end{array}\right)$	$\left(\begin{array}{c} 0.8000\\ 0.2000 \end{array}\right)$	$\left(\begin{array}{c} 0.8000\\ 0.2000 \end{array}\right)$
ℜ_5_	$\left(\begin{array}{c} 0.8000\\ 0.2000 \end{array}\right)$	$\left(\begin{array}{c} 0.7667\\ 0.2333 \end{array}\right)$	$\left(\begin{array}{c} 0.6667\\ 0.3333 \end{array}\right)$	$\left(\begin{array}{c} 0.7000\\ 0.3000 \end{array}\right)$	$\left(\begin{array}{c} 0.7667\\ 0.2333 \end{array}\right)$	$\left(\begin{array}{c} 0.3333\\ 0.6667 \end{array}\right)$	$\left(\begin{array}{c} 0.3333\\ 0.5667 \end{array}\right)$
ℜ_6_	$\left(\begin{array}{c} 0.8667\\ 0.1333 \end{array}\right)$	$\left(\begin{array}{c} 0.8000\\ 0.2000 \end{array}\right)$	$\left(\begin{array}{c} 0.6000\\ 0.4000 \end{array}\right)$	$\left(\begin{array}{c} 0.5000\\ 0.5000 \end{array}\right)$	$\left(\begin{array}{c} 0.5667\\ 0.4333 \end{array}\right)$	$\left(\begin{array}{c} 0.7000\\ 0.3000 \end{array}\right)$	$\left(\begin{array}{c} 0.4000\\ 0.6000 \end{array}\right)$
ℜ_7_	$\left(\begin{array}{c} 0.8000\\ 0.2000 \end{array}\right)$	$\left(\begin{array}{c} 0.6000\\ 0.4000 \end{array}\right)$	$\left(\begin{array}{c} 0.7000\\ 0.3000 \end{array}\right)$	$\left(\begin{array}{c} 0.5333\\ 0.4667 \end{array}\right)$	$\left(\begin{array}{c} 0.4000\\ 0.6000 \end{array}\right)$	$\left(\begin{array}{c} 0.3667\\ 0.6333 \end{array}\right)$	$\left(\begin{array}{c} 0.8333\\ 0.1667 \end{array}\right)$

By using the values of the weights of the insecurity indicators, the WNFFDM in Table 6 is obtained.

**Table 6:** WNFFDM for the security experts.

NCRN	*z*_1_	*z*_2_	*z*_3_	*z*_4_	*z*_5_	*z*_6_	*z*_7_
ℜ_1_	$\left(\begin{array}{c} 0.1870\\ 0.3047 \end{array}\right)$	$\left(\begin{array}{c} 0.2066\\ 0.2938 \end{array}\right)$	$\left(\begin{array}{c} 0.0277\\ 0.7040 \end{array}\right)$	$\left(\begin{array}{c} 0.0035\\ 0.8707 \end{array}\right)$	$\left(\begin{array}{c} 0.0309\\ 0.7016 \end{array}\right)$	$\left(\begin{array}{c} 0.0009\\ 0.9263 \end{array}\right)$	$\left(\begin{array}{c} 0.0934\\ 0.5436 \end{array}\right)$
ℜ_2_	$\left(\begin{array}{c} 0.1563\\ 0.3476 \end{array}\right)$	$\left(\begin{array}{c} 0.1703\\ 0.3424 \end{array}\right)$	$\left(\begin{array}{c} 0.0149\\ 0.7681 \end{array}\right)$	$\left(\begin{array}{c} 0.0035\\ 0.8707 \end{array}\right)$	$\left(\begin{array}{c} 0.0085\\ 0.8208 \end{array}\right)$	$\left(\begin{array}{c} 0.0015\\ 0.9129 \end{array}\right)$	$\left(\begin{array}{c} 0.0096\\ 0.8243 \end{array}\right)$
ℜ_3_	$\left(\begin{array}{c} 0.1870\\ 0.3047 \end{array}\right)$	$\left(\begin{array}{c} 0.1008\\ 0.4611 \end{array}\right)$	$\left(\begin{array}{c} 0.0804\\ 0.5429 \end{array}\right)$	$\left(\begin{array}{c} 0.0529\\ 0.6266 \end{array}\right)$	$\left(\begin{array}{c} 0.0371\\ 0.6785 \end{array}\right)$	$\left(\begin{array}{c} 0.0075\\ 0.8393 \end{array}\right)$	$\left(\begin{array}{c} 0.0022\\ 0.8998 \end{array}\right)$
ℜ_4_	$\left(\begin{array}{c} 0.2066\\ 0.2938 \end{array}\right)$	$\left(\begin{array}{c} 0.1194\\ 0.4248 \end{array}\right)$	$\left(\begin{array}{c} 0.0804\\ 0.5429 \end{array}\right)$	$\left(\begin{array}{c} 0.0746\\ 0.5684 \end{array}\right)$	$\left(\begin{array}{c} 0.0371\\ 0.6785 \end{array}\right)$	$\left(\begin{array}{c} 0.0787\\ 0.5759 \end{array}\right)$	$\left(\begin{array}{c} 0.0781\\ 0.5784 \end{array}\right)$
ℜ_5_	$\left(\begin{array}{c} 0.1316\\ 0.3870 \end{array}\right)$	$\left(\begin{array}{c} 0.1008\\ 0.4611 \end{array}\right)$	$\left(\begin{array}{c} 0.0480\\ 0.6306 \end{array}\right)$	$\left(\begin{array}{c} 0.0529\\ 0.6266 \end{array}\right)$	$\left(\begin{array}{c} 0.0743\\ 0.5696 \end{array}\right)$	$\left(\begin{array}{c} 0.0043\\ 0.8702 \end{array}\right)$	$\left(\begin{array}{c} 0.0043\\ 0.8243 \end{array}\right)$
ℜ_6_	$\left(\begin{array}{c} 0.1870\\ 0.3047 \end{array}\right)$	$\left(\begin{array}{c} 0.1194\\ 0.4248 \end{array}\right)$	$\left(\begin{array}{c} 0.0335\\ 0.6807 \end{array}\right)$	$\left(\begin{array}{c} 0.0171\\ 0.7641 \end{array}\right)$	$\left(\begin{array}{c} 0.0256\\ 0.7237 \end{array}\right)$	$\left(\begin{array}{c} 0.0469\\ 0.6618 \end{array}\right)$	$\left(\begin{array}{c} 0.0075\\ 0.8405 \end{array}\right)$
ℜ_7_	$\left(\begin{array}{c} 0.1316\\ 0.3870 \end{array}\right)$	$\left(\begin{array}{c} 0.0422\\ 0.6142 \end{array}\right)$	$\left(\begin{array}{c} 0.0571\\ 0.6033 \end{array}\right)$	$\left(\begin{array}{c} 0.0211\\ 0.7439 \end{array}\right)$	$\left(\begin{array}{c} 0.0085\\ 0.8208 \end{array}\right)$	$\left(\begin{array}{c} 0.0058\\ 0.8550 \end{array}\right)$	$\left(\begin{array}{c} 0.0934\\ 0.5436 \end{array}\right)$

Next, we compute NIS and PIS from Table 5, and presented the results in Table 7.

**Table 7:** NIS and PIS.

NCRN	*z*_1_	*z*_2_	*z*_3_	*z*_4_	*z*_5_	*z*_6_	*z*_7_
${\overset{˜}{ℜ}}^{⁎-}$	$\left(\begin{array}{c} 0.9000\\ 0.1000 \end{array}\right)$	$\left(\begin{array}{c} 0.9000\\ 0.1000 \end{array}\right)$	$\left(\begin{array}{c} 0.7667\\ 0.2333 \end{array}\right)$	$\left(\begin{array}{c} 0.7667\\ 0.2333 \end{array}\right)$	$\left(\begin{array}{c} 0.7667\\ 0.2333 \end{array}\right)$	$\left(\begin{array}{c} 0.8000\\ 0.2000 \end{array}\right)$	$\left(\begin{array}{c} 0.2667\\ 0.7333 \end{array}\right)$
${\overset{˜}{ℜ}}^{⁎+}$	$\left(\begin{array}{c} 0.8000\\ 0.2000 \end{array}\right)$	$\left(\begin{array}{c} 0.6000\\ 0.4000 \end{array}\right)$	$\left(\begin{array}{c} 0.4667\\ 0.5333 \end{array}\right)$	$\left(\begin{array}{c} 0.3000\\ 0.7000 \end{array}\right)$	$\left(\begin{array}{c} 0.4000\\ 0.6000 \end{array}\right)$	$\left(\begin{array}{c} 0.2000\\ 0.8000 \end{array}\right)$	$\left(\begin{array}{c} 0.8333\\ 0.1667 \end{array}\right)$

Similarly, the weighted NIS and PIS are computed from Table 6, and presented in Table 8.

**Table 8:** Weighted NIS and PIS.

NCRN	*z*_1_	*z*_2_	*z*_3_	*z*_4_	*z*_5_	*z*_6_	*z*_7_
$\sigma_{j}{\overset{˜}{ℜ}}^{⁎-}$	$\left(\begin{array}{c} 0.1316\\ 0.3870 \end{array}\right)$	$\left(\begin{array}{c} 0.0422\\ 0.6142 \end{array}\right)$	$\left(\begin{array}{c} 0.0149\\ 0.7681 \end{array}\right)$	$\left(\begin{array}{c} 0.0035\\ 0.8707 \end{array}\right)$	$\left(\begin{array}{c} 0.0085\\ 0.8208 \end{array}\right)$	$\left(\begin{array}{c} 0.0009\\ 0.9263 \end{array}\right)$	$\left(\begin{array}{c} 0.0934\\ 0.5436 \end{array}\right)$
$\sigma_{j}{\overset{˜}{ℜ}}^{⁎+}$	$\left(\begin{array}{c} 0.2066\\ 0.2938 \end{array}\right)$	$\left(\begin{array}{c} 0.2066\\ 0.2938 \end{array}\right)$	$\left(\begin{array}{c} 0.0804\\ 0.5429 \end{array}\right)$	$\left(\begin{array}{c} 0.0746\\ 0.5684 \end{array}\right)$	$\left(\begin{array}{c} 0.0743\\ 0.5696 \end{array}\right)$	$\left(\begin{array}{c} 0.0787\\ 0.5759 \end{array}\right)$	$\left(\begin{array}{c} 0.0022\\ 0.8998 \end{array}\right)$

Now, we obtain the correlation coefficients between PIS/NIS in Table 7 and ${ℜ}_{i}$ in Table 4 by using the new FFCCM. The results of the computations are as follows:

$$ϒ({\overset{˜}{ℜ}}^{⁎+},{ℜ}_{1})=0.99830,ϒ({\overset{˜}{ℜ}}^{⁎+},{ℜ}_{2})=0.99837,ϒ({\overset{˜}{ℜ}}^{⁎+},{ℜ}_{3})=0.99891,ϒ({\overset{˜}{ℜ}}^{⁎+},{ℜ}_{4})=0.99986,ϒ({\overset{˜}{ℜ}}^{⁎+},{ℜ}_{5})=0.99896,ϒ({\overset{˜}{ℜ}}^{⁎+},{ℜ}_{6})=0.99934,ϒ({\overset{˜}{ℜ}}^{⁎+},{ℜ}_{7})=0.99805.ϒ({\overset{˜}{ℜ}}^{⁎-},{ℜ}_{1})=0.99755,ϒ({\overset{˜}{ℜ}}^{⁎-},{ℜ}_{2})=0.99899,ϒ({\overset{˜}{ℜ}}^{⁎-},{ℜ}_{3})=0.99919,ϒ({\overset{˜}{ℜ}}^{⁎-},{ℜ}_{4})=0.99678,ϒ({\overset{˜}{ℜ}}^{⁎-},{ℜ}_{5})=0.99884,ϒ({\overset{˜}{ℜ}}^{⁎-},{ℜ}_{6})=0.99883,ϒ({\overset{˜}{ℜ}}^{⁎-},{ℜ}_{7})=0.99825.$$

Similarly, the correlation coefficients between weighted PIS/NIS in Table 8 and ${ℜ}_{i}$ in Table 4 are computed, and the results are:

$$ϒ(\sigma {\overset{˜}{ℜ}}^{⁎+},{ℜ}_{1})=0.99477,ϒ(\sigma {\overset{˜}{ℜ}}^{⁎+},{ℜ}_{2})=0.99492,ϒ(\sigma {\overset{˜}{ℜ}}^{⁎+},{ℜ}_{3})=0.99480,ϒ(\sigma {\overset{˜}{ℜ}}^{⁎+},{ℜ}_{4})=0.99441,ϒ(\sigma {\overset{˜}{ℜ}}^{⁎+},{ℜ}_{5})=0.99571,ϒ(\sigma {\overset{˜}{ℜ}}^{⁎+},{ℜ}_{6})=0.99519,ϒ(\sigma {\overset{˜}{ℜ}}^{⁎+},{ℜ}_{7})=0.99869.ϒ(\sigma {\overset{˜}{ℜ}}^{⁎-},{ℜ}_{1})=0.99419,ϒ(\sigma {\overset{˜}{ℜ}}^{⁎-},{ℜ}_{2})=0.99548,ϒ(\sigma {\overset{˜}{ℜ}}^{⁎-},{ℜ}_{3})=0.99341,ϒ(\sigma {\overset{˜}{ℜ}}^{⁎-},{ℜ}_{4})=0.99090,ϒ(\sigma {\overset{˜}{ℜ}}^{⁎-},{ℜ}_{5})=0.99507,ϒ(\sigma {\overset{˜}{ℜ}}^{⁎-},{ℜ}_{6})=0.99339,ϒ(\sigma {\overset{˜}{ℜ}}^{⁎-},{ℜ}_{7})=0.99567.$$

Next, the closeness coefficients of the correlation coefficients are computed for the case without WNFFDM and the WNFFDM case as follows:

$$\Theta_{1}({ℜ}_{1})=0.5002,\Theta_{2}({ℜ}_{2})=0.4998,\Theta_{3}({ℜ}_{3})=0.4999,\Theta_{4}({ℜ}_{4})=0.5008,\Theta_{5}({ℜ}_{5})=0.5000,\Theta_{6}({ℜ}_{6})=0.5001,\Theta_{7}({ℜ}_{7})=0.4999,\sigma \Theta_{1}({ℜ}_{1})=0.5001,\sigma \Theta_{2}({ℜ}_{2})=0.4999,\sigma \Theta_{3}({ℜ}_{3})=0.5003,\sigma \Theta_{4}({ℜ}_{4})=0.5009,\sigma \Theta_{5}({ℜ}_{5})=0.5002,\sigma \Theta_{6}({ℜ}_{6})=0.5005,\sigma \Theta_{7}({ℜ}_{7})=0.5008.$$

The ranking of the closeness coefficients in descending order shows that $\Theta_{4}({ℜ}_{4})$ is the greatest from both of the cases. Hence, the most insecure state in the NCRN is Niger State as represented by ${ℜ}_{4}$. It is essential to note that the region is experiencing a widespread security crises because of the marginal differences between the closeness coefficients.

#### 4.1.5 Sensitivity analysis

Sensitivity analysis is an investigation of how the uncertainty in the outcome of a mathematical model or system may be partitioned and assigned to dissimilar sources of uncertainty in its inputs. This involves calculating sensitivity indices, which measure the influence of an input or set of inputs on the result. It enhances a reliable result-based decision-making. It is achieved by altering model parameters around certain reference values, with the purpose of investigating how minor input affects the model performance. In this instance, the sensitivity analysis is aimed at determining the effect of WNFFDM on the insecurity evaluation.

The correlation coefficients between PIS/NIS and the states ${ℜ}_{i}$, and the weighted PIS/NIS and the states ${ℜ}_{i}$ are presented in Table 9. In addition, the respective closeness coefficients are also presented in the same table.

**Table 9:** Correlation coefficients/closeness coefficients for sensitivity analysis.

Correlation Coefficients	*i* = 1	*i* = 2	*i* = 3	*i* = 4	*i* = 5	*i* = 6	*i* = 7

$ϒ({\overset{˜}{ℜ}}^{⁎+},{ℜ}_{i})$	0.99830	0.99837	0.99891	0.99986	0.99896	0.99934	0.99805
$ϒ(\sigma {\overset{˜}{ℜ}}^{⁎+},{ℜ}_{i})$	0.99477	0.99492	0.99480	0.99441	0.99571	0.99519	0.99869
$ϒ({\overset{˜}{ℜ}}^{⁎-},{ℜ}_{i})$	0.99419	0.99548	0.99341	0.99090	0.99507	0.99339	0.99567
$ϒ(\sigma {\overset{˜}{ℜ}}^{⁎-},{ℜ}_{i})$	0.99755	0.99899	0.99919	0.99678	0.99884	0.99883	0.99825

Closeness Coefficients	*i* = 1	*i* = 2	*i* = 3	*i* = 4	*i* = 5	*i* = 6	*i* = 7

Θ_*i*_(ℜ_*i*_)	0.5002	0.4998	0.4999	0.5008	0.5000	0.5001	0.4999
*σ*Θ_*i*_(ℜ_*i*_)	0.5001	0.4999	0.5003	0.5009	0.5002	0.5005	0.5008

The information in Table 9 shows that the correlation coefficients for the PIS/NIS derivable from the NFFDM and the states ${ℜ}_{i}$ are marginally greater than the correlation coefficients for the WNFFDM case. This proves that the effect of the WNFFDM on the PIS/NIS slightly reduces the precision of the correlation coefficients. In addition, the effect of the WNFFDM on the PIS/NIS makes the derivable closeness coefficients to some extent greater than the closeness coefficients obtained from the PIS/NIS of NFFDM and the states ${ℜ}_{i}$. Furthermore, the closeness coefficients obtained from the PIS/NIS of NFFDM and the states ${ℜ}_{3}$ and ${ℜ}_{7}$ are equal, which is impossible since ${ℜ}_{3}\neq {ℜ}_{7}$. This setback explains the possible impact of WNFFDM on the closeness coefficient.

By ranking the closeness coefficients in Table 9, we obtain Table 10.

**Table 10:** Ranking for sensitivity analysis.

Cases	Ordering	Most Insecure State
NFFDM	ℜ_4_≽ℜ_1_≽ℜ_6_≽ℜ_5_≽ℜ_3_ = ℜ_7_≽ℜ_2_	Niger State
WNFFDM	ℜ_4_≽ℜ_7_≽ℜ_6_≽ℜ_3_≽ℜ_5_≽ℜ_1_≽ℜ_2_	Niger State

The information in Table 10 indicates that the most insecure state in the NCRN is Niger State. Although the interpretation from both cases is identical, the ordering from the NFFDM case cannot be trusted. In fact, it shows that the insecurity problem in Kogi State (${ℜ}_{3}$) is equal to the insecurity problem in FCT (${ℜ}_{7}$). This identical interpretation is not possible since the security concerns of both Kogi State and FCT are distinguishable. This setback with NFFDM proves that the insecurity assessment/evaluation with the WNFFDM is more reliable than the case with NFFDM.

### 4.2 Comparative analyses

**Figure 3:**
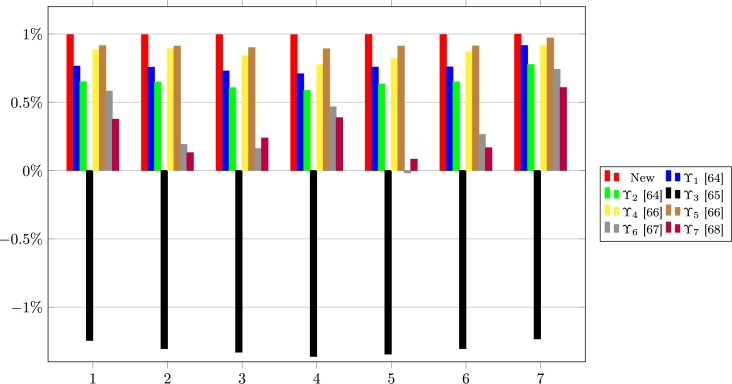
Pictorial of the correlation coefficients between PIS and ℜ_*i*_.

Here, the edge of the new FFCCM over the extant FFCCMs based on the TOPSIS method is shown via comparison in terms of the correlation coefficients, closeness coefficients, and ordering by using the WNFFDM. First, the correlation coefficients, $ϒ(\sigma {\overset{˜}{ℜ}}^{⁎+},{ℜ}_{i})$ and $ϒ(\sigma {\overset{˜}{ℜ}}^{⁎-},{ℜ}_{i})$ are obtained via the new FFCCM and the extant FFCCMs [64], [65], [66], [67], [68] for i=1,2,⋯,K. The results for the computations of $ϒ(\sigma {\overset{˜}{ℜ}}^{⁎+},{ℜ}_{i})$ are presented in Table 11 and Fig. 3.

**Table 11:** Correlation coefficients between PIS and ℜ_*i*_.

FFCCMs	1	2	3	4	5	6	7
ϒ	0.99477	0.99492	0.99480	0.99441	0.99571	0.99519	0.99869
ϒ_1_[64]	0.76464	0.75632	0.72886	0.70843	0.75706	0.75862	0.91513
ϒ_2_[64]	0.64949	0.64680	0.60656	0.58582	0.63334	0.64859	0.77543
ϒ_3_[65]	−1.2435	−1.3031	−1.3296	−1.3601	−1.3432	−1.3036	−1.2322
ϒ_4_[66]	0.88295	0.89195	0.84089	0.77430	0.81947	0.86870	0.91349
ϒ_5_[66]	0.91443	0.91111	0.89994	0.89146	0.91140	0.91203	0.97087
ϒ_6_[67]	0.58194	0.19173	0.16116	0.46690	−0.0166	0.26460	0.74163
ϒ_7_[68]	0.37500	0.13095	0.23810	0.38690	0.08333	0.16667	0.60714

The numbers 1, 2, 3,4,5,6, and 7 represent the correlation coefficients between $\sigma {\overset{˜}{ℜ}}^{⁎+}$ and each of the states, ${ℜ}_{1}$, ${ℜ}_{2}$, ${ℜ}_{3}$, ${ℜ}_{4}$, ${ℜ}_{5}$, ${ℜ}_{6}$, and ${ℜ}_{7}$.

**Figure 4:**
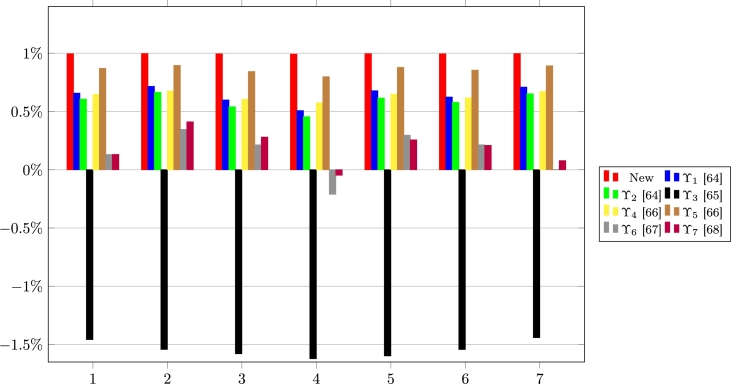
Pictorial of the correlation coefficients between NIS and ℜ_*i*_.

The results for the computations of $ϒ(\sigma {\overset{˜}{ℜ}}^{⁎-},{ℜ}_{i})$ are presented in Table 12 and Fig. 4.

**Table 12:** Correlation coefficients between NIS and ℜ_*i*_.

FFCCMs	1	2	3	4	5	6	7
ϒ	0.99419	0.99548	0.99341	0.99090	0.99507	0.99339	0.99567
ϒ_1_[64]	0.65686	0.71510	0.59818	0.50705	0.67699	0.62309	0.70843
ϒ_2_[64]	0.60528	0.66344	0.54005	0.45487	0.61441	0.57791	0.65122
ϒ_3_[65]	−1.4567	−1.5414	−1.5786	−1.6209	−1.5974	−1.5421	−1.4407
ϒ_4_[66]	0.64605	0.67501	0.60372	0.57339	0.64737	0.61632	0.67072
ϒ_5_[66]	0.86928	0.89424	0.84258	0.79742	0.87807	0.85412	0.89145
ϒ_6_[67]	0.13035	0.34567	0.21261	−0.2111	0.29587	0.21349	0.00229
ϒ_7_[68]	0.13095	0.41071	0.27976	−0.0476	0.25595	0.20833	0.07738

The numbers 1, 2, 3,4,5,6, and 7 represent the correlation coefficients between $\sigma {\overset{˜}{ℜ}}^{⁎-}$ and each of the states, ${ℜ}_{1}$, ${ℜ}_{2}$, ${ℜ}_{3}$, ${ℜ}_{4}$, ${ℜ}_{5}$, ${ℜ}_{6}$, and ${ℜ}_{7}$.

From the results in Table 11, Table 12, and Figure 3, Figure 4, it is certain that the new FFCCM is the most appropriate and reliable approach of FFCC. Again, it is observed that the FFCCM in [65] is the most inappropriate FFCCM because it yields results that exceed the FFCC range of values.

**Figure 5:**
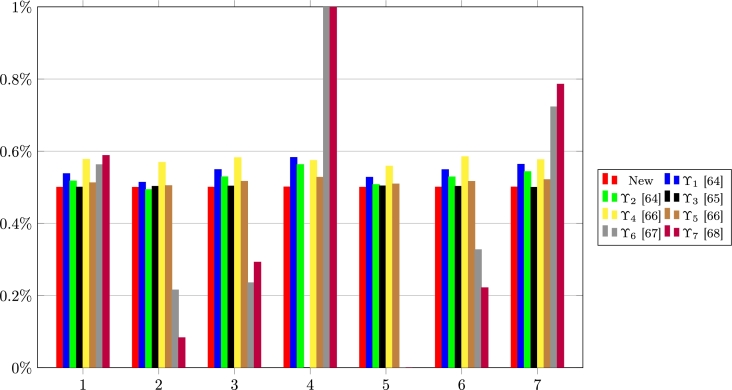
Charts of the closeness coefficients.

Next, we compute comparative $\sigma \Theta_{i}({ℜ}_{i})$ for i=1,2,⋯,7. The closeness coefficients via the FFCCMs are given in Table 13 and shown in Fig. 5.

**Table 13:** Closeness coefficients for ℜ_*i*_.

PFCCMs	*σ*Θ_1_(ℜ_1_)	*σ*Θ_2_(ℜ_2_)	*σ*Θ_3_(ℜ_3_)	*σ*Θ_4_(ℜ_4_)	*σ*Θ_5_(ℜ_5_)	*σ*Θ_6_(ℜ_6_)	*σ*Θ_7_(ℜ_7_)
ϒ	0.5001	0.4999	0.5003	0.5009	0.5002	0.5005	0.5008
ϒ_1_[64]	0.5379	0.5140	0.5492	0.5828	0.5279	0.5490	0.5637
ϒ_2_[64]	0.5176	0.4937	0.5290	0.5629	0.5076	0.5288	0.5435
ϒ_3_[65]	0.5005	0.5027	0.5036	0.0000	0.5040	0.5027	0.5000
ϒ_4_[66]	0.5775	0.5692	0.5821	0.5745	0.5587	0.5850	0.5766
ϒ_5_[66]	0.5127	0.5047	0.5165	0.5278	0.5093	0.5164	0.5213
ϒ_6_[67]	0.5628	0.2155	0.2355	1.0000	0.0000	0.3272	0.7229
ϒ_7_[68]	0.5883	0.0833	0.2926	1.0000	0.0000	0.2217	0.7857

Fig. 5 shows the reliability of the new FFCCM because it yields the least-consistent closeness coefficients. For proper interpretation, we use the information in Table 13 to present the closeness coefficients' ordering in Table 14.

**Table 14:** Insecurity analysis.

FFCCMs	Ordering	Most Insecure State
ϒ	ℜ_4_ ≻ ℜ_7_ ≻ ℜ_6_ ≻ ℜ_3_ ≻ ℜ_5_ ≻ ℜ_1_ ≻ ℜ_2_	ℜ_4_
ϒ_1_[64]	ℜ_4_ ≻ ℜ_7_ ≻ ℜ_3_ ≻ ℜ_6_ ≻ ℜ_1_ ≻ ℜ_5_ ≻ ℜ_2_	ℜ_4_
ϒ_2_[64]	ℜ_4_ ≻ ℜ_7_ ≻ ℜ_3_ ≻ ℜ_6_ ≻ ℜ_5_ ≻ ℜ_5_ ≻ ℜ_7_	ℜ_4_
ϒ_3_[65]	ℜ_5_ ≻ ℜ_3_ ≻ ℜ_2_ = ℜ_6_ ≻ ℜ_1_ ≻ ℜ_7_ ≻ ℜ_4_	ℜ_5_
ϒ_4_[66]	ℜ_6_ ≻ ℜ_3_ ≻ ℜ_1_ ≻ ℜ_7_ ≻ ℜ_4_ ≻ ℜ_2_ ≻ ℜ_5_	ℜ_6_
ϒ_5_[66]	ℜ_4_ ≻ ℜ_7_ ≻ ℜ_3_ ≻ ℜ_6_ ≻ ℜ_1_ ≻ ℜ_5_ ≻ ℜ_2_	ℜ_4_
ϒ_6_[67]	ℜ_4_ ≻ ℜ_7_ ≻ ℜ_3_ ≻ ℜ_6_ ≻ ℜ_3_ ≻ ℜ_2_ ≻ ℜ_5_	ℜ_4_
ϒ_7_[68]	ℜ_4_ ≻ ℜ_7_ ≻ ℜ_1_ ≻ ℜ_3_ ≻ ℜ_6_ ≻ ℜ_2_ ≻ ℜ_5_	ℜ_4_

The information in Table 14 shows that the most insecure state in the NCRN is Niger State. It is observed that the FFCCMs ${ϒ}_{3}$ [65] and ${ϒ}_{4}$ [66] give otherwise interpretation, but their interpretations cannot be trusted because of their limitations. The information from the FFCCMs in [64], [66] and the new FFCCM show that the region is not secured because the closeness coefficients are very close, and so intended tourists should be security cautious. The information with regards to closeness coefficients deduced from the FFCCMs in [65], [67], [68] is very misleading and should be discarded.

## 5 Conclusion

This paper presented a novel FFCCM for an effective computation of correlation coefficient between FFSs and discussed its application in the assessment and evaluation of security crises in the NCRN. A reliable insecurity assessment is very difficult to obtain because of the imprecision of security crises. Based on Spearman's correlation coefficient, a novel FFCCM was created to eliminate any potential error that might prevent a reliable security decision. To justify the need for a new FFCCM, a number of existing FFCCMs were analyzed and their shortcomings were identified. Due to the shortcomings, a new FFCCM was constructed, and how the new FFCCM addressed the issues observed with the existing FFCCMs was demonstrated. In addition, the new FFCCM satisfied the FFCC requirements and its validity was confirmed based on theoretical results. Furthermore, the novel FFCCM was used to address the problem of insecurity assessment in the NCRN. The data for the evaluation and assessment was collected using LVs from three security experts with an in-depth knowledge of the security concerns in the region, and the LVs were transformed to FFNs for the assessment. The finding from the evaluation shows the widespread nature of insecurity within the NCRN with Niger State as the insecure hotbed. In order to show the inherent value of the new FFCCM over the existing ones, we compared the new FFCCM with some existing FFCCMs [64], [65], [66], [67], [68]. The comparative analyses show the superiority of the new FFCCM in terms of reliability, precision, and suitability. The fuzziness that comes with the process of insecurity assessment was effectively managed by the Fermatean fuzzy security assessment approach. The new FFCCM, however, might only be useful in situations where the sum of ${\text{MD}}^{3}$ and ${\text{NMD}}^{3}$ is at most one. In addition, the application of the new FFCCM is limited due to its inability to model some complex decision problems in environments like p,q-quasirung fuzzy sets, q-rung orthopair fuzzy sets, spherical fuzzy set, etc. For additional research, the new FFCCM could be examined in complex fuzzy settings with some modifications and used to discuss decision-making problems under q-rung orthopair fuzzy sets [74], [75], [76], quasirung fuzzy sets and 3,4-quasirung orthopair fuzzy sets [77], [78]. Finally, the novel Fermatean fuzzy security assessment could be combined with qualitative and quantitative risk analysis techniques to enhance better security assessment in future investigation.

## CRediT authorship contribution statement

**Paul Augustine Ejegwa:** Validation, Software, Methodology, Data curation, Conceptualization. **Nasreen Kausar:** Writing – original draft, Validation, Formal analysis, Data curation, Conceptualization. **Nezir Aydin:** Writing – review & editing, Writing – original draft, Validation, Supervision, Investigation, Conceptualization. **Yuming Feng:** Writing – review & editing, Validation, Conceptualization. **Oludolapo Akanni Olanrewaju:** Writing – review & editing, Methodology, Investigation, Funding acquisition, Conceptualization.

## Declaration of Competing Interest

The authors declare that they have no known competing financial interests or personal relationships that could have appeared to influence the work reported in this paper.

## Data Availability

Data included in the article/supplementary material is referenced in the article.

## Appendix A

### Proof of Theorem 1

Proof The FFCC conditions are enumerated as follows:

(i) 0≤ϒ(ℑ,ℜ)≤1,
(ii) ϒ(ℑ,ℜ)=1 iff ℑ=ℜ,
(iii) ϒ(ℑ,ℜ)=ϒ(ℜ,ℑ).

Now, we prove 0≤ϒ(ℑ,ℜ)≤1. But, since

$${\left[\max\{{ℑ}_{m}^{3}(z_{j}),{ℜ}_{m}^{3}(z_{j})\}-\min\{{ℑ}_{m}^{3}(z_{j}),{ℜ}_{m}^{3}(z_{j})\}\right]}^{3}\geq 0,{\left[\max\{{ℑ}_{n}^{3}(z_{j}),{ℜ}_{n}^{3}(z_{j})\}-\min\{{ℑ}_{n}^{3}(z_{j}),{ℜ}_{n}^{3}(z_{j})\}\right]}^{3}\geq 0,{\left[\max\{{ℑ}_{h}^{3}(z_{j}),{ℜ}_{h}^{3}(z_{j})\}-\min\{{ℑ}_{h}^{3}(z_{j}),{ℜ}_{h}^{3}(z_{j})\}\right]}^{3}\geq 0,$$

then ϒ(ℑ,ℜ)≥0. Assume that

$$6\sum_{j=1}^{N}{\left[\max\{{ℑ}_{m}^{3}(z_{j}),{ℜ}_{m}^{3}(z_{j})\}-\min\{{ℑ}_{m}^{3}(z_{j}),{ℜ}_{m}^{3}(z_{j})\}\right]}^{3}=\Omega_{1},6\sum_{j=1}^{N}{\left[\max\{{ℑ}_{n}^{3}(z_{j}),{ℜ}_{n}^{3}(z_{j})\}-\min\{{ℑ}_{n}^{3}(z_{j}),{ℜ}_{n}^{3}(z_{j})\}\right]}^{3}=\Omega_{2},6\sum_{j=1}^{N}{\left[\max\{{ℑ}_{h}^{3}(z_{j}),{ℜ}_{h}^{3}(z_{j})\}-\min\{{ℑ}_{h}^{3}(z_{j}),{ℜ}_{h}^{3}(z_{j})\}\right]}^{3}=\Omega_{3}.$$

Then,

$$ϒ(ℑ,ℜ)=1-\frac{6\sum_{j=1}^{N}}{3({\left(N+1\right)}^{3}-(N+1))}\left\{\begin{array}{c} {\left[\max\{{ℑ}_{m}^{3}(z_{j}),{ℜ}_{m}^{3}(z_{j})\}-\min\{{ℑ}_{m}^{3}(z_{j}),{ℜ}_{m}^{3}(z_{j})\}\right]}^{3}\\ +{\left[\max\{{ℑ}_{n}^{3}(z_{j}),{ℜ}_{n}^{3}(z_{j})\}-\min\{{ℑ}_{n}^{3}(z_{j}),{ℜ}_{n}^{3}(z_{j})\}\right]}^{3}\\ +{\left[\max\{{ℑ}_{h}^{3}(z_{j}),{ℜ}_{h}^{3}(z_{j})\}-\min\{{ℑ}_{h}^{3}(z_{j}),{ℜ}_{h}^{3}(z_{j})\}\right]}^{3} \end{array}\right\}\leq 1-\frac{1}{3({\left(N+1\right)}^{3}-(N+1))}\left\{\begin{array}{c} 6\sum_{j=1}^{N}{\left[\max\{{ℑ}_{m}^{3}(z_{j}),{ℜ}_{m}^{3}(z_{j})\}-\min\{{ℑ}_{m}^{3}(z_{j}),{ℜ}_{m}^{3}(z_{j})\}\right]}^{3}\\ +6\sum_{j=1}^{N}{\left[\max\{{ℑ}_{n}^{3}(z_{j}),{ℜ}_{n}^{3}(z_{j})\}-\min\{{ℑ}_{n}^{3}(z_{j}),{ℜ}_{n}^{3}(z_{j})\}\right]}^{3}\\ +6\sum_{j=1}^{N}{\left[\max\{{ℑ}_{h}^{3}(z_{j}),{ℜ}_{h}^{3}(z_{j})\}-\min\{{ℑ}_{h}^{3}(z_{j}),{ℜ}_{h}^{3}(z_{j})\}\right]}^{3} \end{array}\right\}=1-\frac{\left(\Omega_{1}+\Omega_{2}+\Omega_{3}\right)}{3({\left(N+1\right)}^{3}-(N+1))}.$$

Thus,

$$ϒ(ℑ,ℜ)-1=-\frac{\left(\Omega_{1}+\Omega_{2}+\Omega_{3}\right)}{3({\left(N+1\right)}^{3}-(N+1))}\leq 0,$$

and so ϒ(ℑ,ℜ)≤1. Since ϒ(ℑ,ℜ)≥0 and ϒ(ℑ,ℜ)≤1, we have 0≤ϒ(ℑ,ℜ)≤1, which proves (i). Assume ϒ(ℑ,ℜ)=1, then we have

$$\frac{6\sum_{j=1}^{N}}{3({\left(N+1\right)}^{3}-(N+1))}\left\{\begin{array}{c} {\left[\max\{{ℑ}_{m}^{3}(z_{j}),{ℜ}_{m}^{3}(z_{j})\}-\min\{{ℑ}_{m}^{3}(z_{j}),{ℜ}_{m}^{3}(z_{j})\}\right]}^{3}\\ +{\left[\max\{{ℑ}_{n}^{3}(z_{j}),{ℜ}_{n}^{3}(z_{j})\}-\min\{{ℑ}_{n}^{3}(z_{j}),{ℜ}_{n}^{3}(z_{j})\}\right]}^{3}\\ +{\left[\max\{{ℑ}_{h}^{3}(z_{j}),{ℜ}_{h}^{3}(z_{j})\}-\min\{{ℑ}_{h}^{3}(z_{j}),{ℜ}_{h}^{3}(z_{j})\}\right]}^{3} \end{array}\right\}=0,$$

i.e.,

$${\left[\max\{{ℑ}_{m}^{3}(z_{j}),{ℜ}_{m}^{3}(z_{j})\}-\min\{{ℑ}_{m}^{3}(z_{j}),{ℜ}_{m}^{3}(z_{j})\}\right]}^{3}=0,{\left[\max\{{ℑ}_{n}^{3}(z_{j}),{ℜ}_{n}^{3}(z_{j})\}-\min\{{ℑ}_{n}^{3}(z_{j}),{ℜ}_{n}^{3}(z_{j})\}\right]}^{3}=0,{\left[\max\{{ℑ}_{h}^{3}(z_{j}),{ℜ}_{h}^{3}(z_{j})\}-\min\{{ℑ}_{h}^{3}(z_{j}),{ℜ}_{h}^{3}(z_{j})\}\right]}^{3}=0.$$

Thus, ${ℑ}_{m}(z_{j})={ℜ}_{m}(z_{j})$, ${ℑ}_{n}(z_{j})={ℜ}_{n}(z_{j})$ and ${ℑ}_{h}(z_{j})={ℜ}_{h}(z_{j})$. Hence, ℑ=ℜ. Conversely, suppose ℑ=ℜ. Then, we get

$$\frac{6\sum_{j=1}^{N}}{3({\left(N+1\right)}^{3}-(N+1))}\left\{\begin{array}{c} {\left[\max\{{ℑ}_{m}^{3}(z_{j}),{ℜ}_{m}^{3}(z_{j})\}-\min\{{ℑ}_{m}^{3}(z_{j}),{ℜ}_{m}^{3}(z_{j})\}\right]}^{3}\\ +{\left[\max\{{ℑ}_{n}^{3}(z_{j}),{ℜ}_{n}^{3}(z_{j})\}-\min\{{ℑ}_{n}^{3}(z_{j}),{ℜ}_{n}^{3}(z_{j})\}\right]}^{3}\\ +{\left[\max\{{ℑ}_{h}^{3}(z_{j}),{ℜ}_{h}^{3}(z_{j})\}-\min\{{ℑ}_{h}^{3}(z_{j}),{ℜ}_{h}^{3}(z_{j})\}\right]}^{3} \end{array}\right\}=0,$$

and hence ϒ(ℑ,ℜ)=1, which shows (ii). Finally, (iii) follows because

$$ϒ(ℑ,ℜ)==\frac{1}{3}\left\{\begin{array}{c} 1-\frac{6\sum_{j=1}^{N}{\left[\max\{{ℑ}_{m}^{3}(z_{j}),{ℜ}_{m}^{3}(z_{j})\}-\min\{{ℑ}_{m}^{3}(z_{j}),{ℜ}_{m}^{3}(z_{j})\}\right]}^{3}}{{\left(N+1\right)}^{3}-(N+1)}+\\ 1-\frac{6\sum_{j=1}^{N}{\left[\max\{{ℑ}_{n}^{3}(z_{j}),{ℜ}_{n}^{3}(z_{j})\}-\min\{{ℑ}_{n}^{3}(z_{j}),{ℜ}_{n}^{3}(z_{j})\}\right]}^{3}}{{\left(N+1\right)}^{3}-(N+1)}+\\ 1-\frac{6\sum_{j=1}^{N}{\left[\max\{{ℑ}_{h}^{3}(z_{j}),{ℜ}_{h}^{3}(z_{j})\}-\min\{{ℑ}_{h}^{3}(z_{j}),{ℜ}_{h}^{3}(z_{j})\}\right]}^{3}}{{\left(N+1\right)}^{3}-(N+1)} \end{array}\right\}=\frac{1}{3}\left\{\begin{array}{c} \frac{{\left(N+1\right)}^{3}-(N+1)-6\sum_{j=1}^{N}{\left[\max\{{ℑ}_{m}^{3}(z_{j}),{ℜ}_{m}^{3}(z_{j})\}-\min\{{ℑ}_{m}^{3}(z_{j}),{ℜ}_{m}^{3}(z_{j})\}\right]}^{3}}{{\left(N+1\right)}^{3}-(N+1)}\\ +\frac{{\left(N+1\right)}^{3}-(N+1)-6\sum_{j=1}^{N}{\left[\max\{{ℑ}_{n}^{3}(z_{j}),{ℜ}_{n}^{3}(z_{j})\}-\min\{{ℑ}_{n}^{3}(z_{j}),{ℜ}_{n}^{3}(z_{j})\}\right]}^{3}}{{\left(N+1\right)}^{3}-(N+1)}\\ +\frac{{\left(N+1\right)}^{3}-(N+1)-6\sum_{j=1}^{N}{\left[\max\{{ℑ}_{h}^{3}(z_{j}),{ℜ}_{h}^{3}(z_{j})\}-\min\{{ℑ}_{h}^{3}(z_{j}),{ℜ}_{h}^{3}(z_{j})\}\right]}^{3}}{{\left(N+1\right)}^{3}-(N+1)} \end{array}\right\}=\frac{1}{3}\left\{\begin{array}{c} \frac{{\left(N+1\right)}^{3}-(N+1)-6\sum_{j=1}^{N}{\left[\max\{{ℜ}_{m}^{3}(z_{j}),{ℑ}_{m}^{3}(z_{j})\}-\min\{{ℜ}_{m}^{3}(z_{j}),{ℑ}_{m}^{3}(z_{j})\}\right]}^{3}}{{\left(N+1\right)}^{3}-(N+1)}\\ +\frac{{\left(N+1\right)}^{3}-(N+1)-6\sum_{j=1}^{N}{\left[\max\{{ℜ}_{n}^{3}(z_{j}),{ℑ}_{n}^{3}(z_{j})\}-\min\{{ℜ}_{n}^{3}(z_{j}),{ℑ}_{n}^{3}(z_{j})\}\right]}^{3}}{{\left(N+1\right)}^{3}-(N+1)}\\ +\frac{{\left(N+1\right)}^{3}-(N+1)-6\sum_{j=1}^{N}{\left[\max\{{ℜ}_{h}^{3}(z_{j}),{ℑ}_{h}^{3}(z_{j})\}-\min\{{ℜ}_{h}^{3}(z_{j}),{ℑ}_{h}^{3}(z_{j})\}\right]}^{3}}{{\left(N+1\right)}^{3}-(N+1)} \end{array}\right\}=ϒ(ℜ,ℑ).$$

Hence, ϒ(ℑ,ℜ) fulfilled the conditions of FFCC. □

### Proof of Theorem 2

Proof Analogous to Theorem 1. □
